# metilene^3^: identifying DMRs across multiple conditions with auto-classification

**DOI:** 10.1038/s41467-026-74931-y

**Published:** 2026-07-04

**Authors:** Zhihan Zhu, Stephan H. Bernhart, Frank Jühling, Helene Kretzmer, Steve Hoffmann

**Affiliations:** 1 Max Planck Institute for Molecular Genetics, Berlin, Germany; 2 Department of Mathematics and Computer Science, Free University Berlin, Berlin, Germany; 3 LeiCeM - Leipzig Center of Metabolism, Leipzig University, Leipzig, Germany; 4 University of Strasbourg, Inserm, Institute for Translational Medicine and Liver Disease (ITM), UMR_S1110, Strasbourg, France; 5 Hasso Plattner Institute for Digital Engineering, Digital Engineering Faculty, University of Potsdam, Potsdam, Germany; 6 Hoffmann Group, Leibniz Institute on Aging - Fritz Lipmann Institute (FLI), Jena, Germany

**Keywords:** Software, Data processing

## Abstract

DNA methylation is a critical epigenetic mark across numerous species, and identifying differentially methylated regions (DMRs) is essential for understanding genome regulation. Most existing DMR detection methods require predefined sample conditions, limiting the discovery of new epigenetic patterns, especially when group identities are unknown or uncertain, as is common in clinical settings. Additionally, only a very few approaches enable comparisons across multiple conditions. To address this significant gap, we present metilene^3^, a method for rapid, multi-condition DMR detection that operates in both supervised and unsupervised modes, using user-provided labels or autonomously clustering unlabeled samples. By segmenting the genome based on multiple pairwise methylation difference signals, metilene^3^ enables sample classification and DMR-anchored inference of epigenetic relationships. Using simulated and diverse human datasets, we show that metilene^3^ accurately detects DMRs, robustly clusters samples, and holds the potential to reveal new regulatory elements and sample stratifications. Specifically, in a pancreatic tissue dataset, metilene^3^ identifies DMRs enriched for key transcription factors involved in pancreatic cancer development, hinting towards an altered NFKB-NFAT regulatory program. Together, metilene^3^ provides a fast, interpretable framework for exploring heterogeneous methylomes and discovering epigenetic patterns across complex biological and clinical datasets.

## Abstract (web summary)

Differentially methylated regions (DMRs) play a central role in development and disease. Here, the authors present metilene^3^, a methylation analysis tool that identifies DMRs in multi-group and unsupervised settings, enabling the discovery of biologically meaningful sample groups from DMR profiles.

## Introduction

The methylation of the 5-carbon of cytosines (5mC) is a heritable epigenetic mark that is maintained through cell division and plays a crucial role in shaping cell identity and developmental state, independent of the underlying DNA sequence. In mammals, where cytosine methylation predominantly occurs in CpG contexts, it is a key indicator of various regulatory processes, including those involved in development, aging, and disease^1^. In cancer, for instance, DNA methylation patterns often shift, including global hypomethylation and localized promoter hypermethylation^2^. These aberrant patterns can be involved in silencing tumor suppressor genes or activating oncogenic pathways, contributing to disease initiation and progression, such as methylation of *CCND2* (cyclin D2) in pancreatic cancer^3^ and epigenetic alterations in IDH-mutant glioma^4^. Thus, examining DNA methylation differences yields valuable insights into biological processes and offers opportunities for biomarker discovery across various conditions. A key aspect of such studies is the identification of differentially methylated CpGs and differentially methylated regions (DMRs). However, available DMR callers and pipelines, including metilene (ref. ^5^.), typically require that samples be categorized, i.e., assigned to a specific condition^5–11^. Detecting DMRs across multiple, potentially unknown groups, whether supervised or unsupervised, remains a critical challenge. Furthermore, as the number of large-scale methylation studies grows, the systematic identification of biologically relevant DMRs is becoming increasingly complex, underscoring the need for more versatile and efficient analytical approaches.

Methods such as SMART2^12^ and wgbstools^13^ have been developed to facilitate multi-condition comparisons. In brief, SMART2 leverages Shannon entropy to identify CpGs and regions that are specific to given cell types^12^, and wgbstools segments the genome by minimizing methylation variance of neighboring CpGs. Subsequently, group-specific patterns are identified through a one-versus-all comparison of mean segment methylation^13^. Recently, two methods for group-agnostic detection of differential methylation were presented. One of them, Aclust2.0^14^, uses Illumina methylation arrays, and the other, Methylscore^15^, is specifically designed for plants and provides unsupervised DMR detection along with local clustering based on a hidden Markov model, without global grouping. However, many studies require a flexible framework, i.e., one that can identify methylation differences without relying solely on predefined conditions while allowing downstream classification of samples. This is especially important for identifying novel subgroups, such as disease subtypes, to improve diagnosis, outcome prediction, or even treatment^16,17^. To date, most clustering methods used for this task select CpGs with high variability^18^, such as K-means or hierarchical clustering. Such selection processes may substantially limit biological interpretability, for example, by isolating CpGs from their CpG neighborhoods and removing CpGs with consistent but subtle methylation changes.

Metilene^3^ closes this methodological gap. Building on the core of its predecessor, it utilizes new algorithms to enable unsupervised, label-free DMR detection and sample classification, supervised multi-group comparisons, and systematic analysis of the inferred DMRs. Implementing a multi-sample version of the circular binary segmentation (CBS) algorithm, metilene^3^ independently classifies the samples at individual genomic locations (local classification). The DMRs and local classifications are used to perform divisive clustering of samples to construct a binary linkage tree (Differentially Methylated Tree, DMTree) that reflects epigenetic relationships among the input samples. This step is specifically relevant to distinguish recurring patterns in sample similarity from noise or confounding factor-dependent groupings. Thus, metilene^3^ automatically self-corrects the unsupervised sample clustering by focusing on robust patterns. Using simulated and biological data, we show that metilene^3^ is sensitive, accurate, and fast, enabling DMR calling on a standard laptop even across multiple conditions. By reanalyzing public datasets, we demonstrate metilene^3^’s ability to uncover relevant insights into cell differentiation and cancer biology, highlighting the value of revisiting existing data with advanced DNA methylation analysis tools.

## Results

### Algorithm

#### A tool for studying methylomes across multiple conditions

**Fig. 1:**
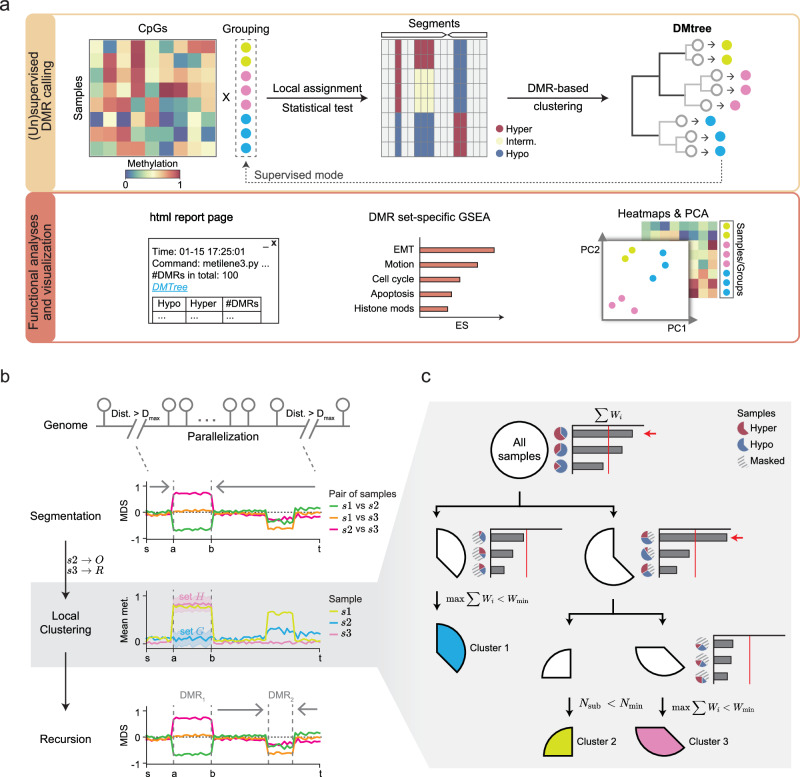
Metilene^3^ enables unsupervised and supervised detection of differentially methylated regions. **a** Overview of the metilene^3^ workflow. (Top) Metilene^3^ operates in two modes: an unsupervised mode and a supervised mode. In the unsupervised mode, only a CpG × sample methylation matrix is required as input. In the supervised mode, an additional label assignment of samples to conditions must be provided. Both modes identify differentially methylated regions (DMRs). In the unsupervised mode, these DMRs are used to construct differentially methylated trees (DMTrees) for clustering and inferring sample relationships. (Bottom) Metilene^3^ offers various downstream analysis functions, including visualization tools and Gene Set Enrichment Analysis (GSEA). **b** Multi-group DMR identification algorithm in metilene^3^. The genome is first pre-segmented based on CpG distances. Next, a modified circular binary segmentation algorithm is applied to identify optimal subregions across all pairwise group comparisons, and the one resulting in the maximized Z-scores (sample $O$ and $R$) is selected as a potential DMR. In such a subregion, local distance-based clustering assigns samples to the pair of groups that yielded the highest Z-score. Samples are then categorized as set H (hypermethylated, red), set G (hypomethylated, blue), or set I (intermediate, if no unambiguous assignment is possible). The recursive segmentation process continues until no additional subregions meeting the predefined criteria can be identified. **c** DMTree inference algorithm in metilene^3^. Metilene^3^ constructs a binary tree of samples by recursively splitting them based on DMR patterns with the highest sum of weight score ($\sum {W}_{i}$). The final clusters (colored) are defined by nodes that meet the following criteria: (1) the number of samples in the cluster (${N}_{{sub}}$) exceeds a minimum number (${N}_{\min }$); and (2) the sum of weight score of the DMR pattern ($\sum {W}_{i}$) exceeds a predefined threshold (${W}_{\min }$). This hierarchical clustering approach enables the identification of methylation-driven sample groupings.

Metilene^3^ introduces two key advancements: (1) It enables supervised multi-group comparisons alongside traditional two-way analyses, and (2) features an unsupervised mode, allowing DMR detection without user-supplied sample categories. To achieve this, metilene^3^ augments DMR detection with a local classification step, enabling the construction of a binary cluster tree (DMTree) based on recurrent sample-by-classification patterns across DMRs. This approach aims to reduce sensitivity to noise and batch effects by using recurrent group configurations with substantial methylation differences, thereby facilitating the discovery of robust, potentially novel sample subgroups and enabling the exploration of sample- or subgroup-specific alterations, which are then used for supervised DMR detection. Key parameters and their default values are described in the Methods. Additionally, a summary report supports data visualization and downstream processing, including figure generation, DMR annotation to the nearest genes, and gene set enrichment analysis (Fig. 1a).

For DMR detection between two or more groups (or samples in unsupervised mode), metilene^3^ employs the originally introduced circular binary segmentation (CBS)^5^ of mean methylation difference signals (MDSs) (Fig. 1b). Since the unsupervised mode represents the more general case, we first describe metilene^3^’s approach in this context.

#### Circular binary segmentation and local sample classification in the unsupervised mode

For all pairs of samples in some segment $$[s,t]$$, the Z-score for MDS is maximized subject to the genomic coordinates $$[u,v]$$ with $$s\le u < v\le t$$. The pair of samples $$(O,R)$$ and the segment $$[a,b]$$ maximizing the Z-score are further processed. In $$[a,b]$$, one of the samples is hypermethylated (or hypomethylated) compared to the other. Therefore, the conditions $$O$$ and $$R$$ are chosen as the first elements of the sets $$G$$ and $$H$$, respectively. Next, all other samples are assigned to $$G$$ or $$H$$ based on the minimum methylation distance to $$O$$ and $$R$$ in $$[a,b]$$ (see **Methods**). If an unambiguous classification is impossible, samples are assigned to an ambiguous, intermediate group (Fig. 1b), suggesting they may, for example, be in a transition state and exhibit a heterogeneous methylation pattern across the underlying cells, resulting in subtle methylation differences compared to both the hypo- and hypermethylated groups within this region. We refer to this procedure as *local* sample classification because a sample is assigned to one of the three sets based solely on the methylation values in $$[a,b]$$. Subsequently, metilene^3^ performs a two-dimensional KS test between $$G$$ and $$H$$ for all methylation values within $$[a,b]$$. Based on the test result and additional criteria such as the number of CpGs, the final segment is accepted or rejected as a DMR. If accepted, the recursion proceeds with the segment $$[s,a)$$ and $$(b,t]$$. Otherwise, the recursion terminates for the segment $$[s,t]$$.

#### Construction of DMTrees

Upon completion of the CBS, the identified DMRs and their corresponding local sample classifications are used to construct the DMTree (Fig. 1c). Conceptually, for a given set of samples, metilene^3^ identifies recurring local classification patterns, i.e., sample assignments to $G$, $H$, and $I$ that are similar across multiple DMRs. To determine the most impactful classification in each step of the tree construction, metilene^3^ calculates $\sum {W}_{i}$, the total methylation difference of a given pattern, defined as the sum of the mean MDS values for all pattern-associated DMRs.

The DMTree construction begins by considering all samples. The classification pattern with the largest $\sum {W}_{i}$-value is placed at the root, and samples are then split into hypomethylated and hypermethylated groups, forming the first two branches of the DMTree. Samples in $I$ are temporarily assigned to the smaller set to avoid the split being driven by group size (see **Methods**). For each branch, the $\sum {W}_{i}$-values are recalculated based on the subset of samples assigned to that branch. Subsequently, the pattern with the highest $\sum {W}_{i}$ determines the next split. This process is repeated until a single sample is left, i.e., a leaf node is reached. Samples belonging to branches that undercut ${{\rm{W}}_{\min}}$ will be jointly assigned to one of the metilene^3^-reported clusters. After the final clustering, metilene^3^ runs the DMR detection again, now in supervised mode, using the clusters as groups to identify more DMRs (Fig. 1a).

#### Supervised multi-group DMR calling

In the supervised mode, for some segment $[s,t]$, metilene^3^ also seeks to maximize the Z-score for the MDSs subject to the genomic coordinates $[u,v]$ with $s\le u < v\le t$. In contrast to the unsupervised mode, however, the Z-scores are calculated for all pairs of groups rather than individual samples. The subsequent steps are identical to the calculations in the unsupervised case.

Of note, a major computational challenge of the outlined calculations is the quadratic increase in MDSs as the number of conditions (in supervised mode) or samples (in unsupervised mode with automated sample classification) grows (Suppl. Fig. 1a). However, in both modes, the genome is pre-segmented whenever the distance between two CpG sites becomes too large. All such segments can be analyzed independently, enabling full parallelization of the approach (Fig. 1b).

### Benchmarking

#### Performance tests on simulated data

**Fig. 2:**
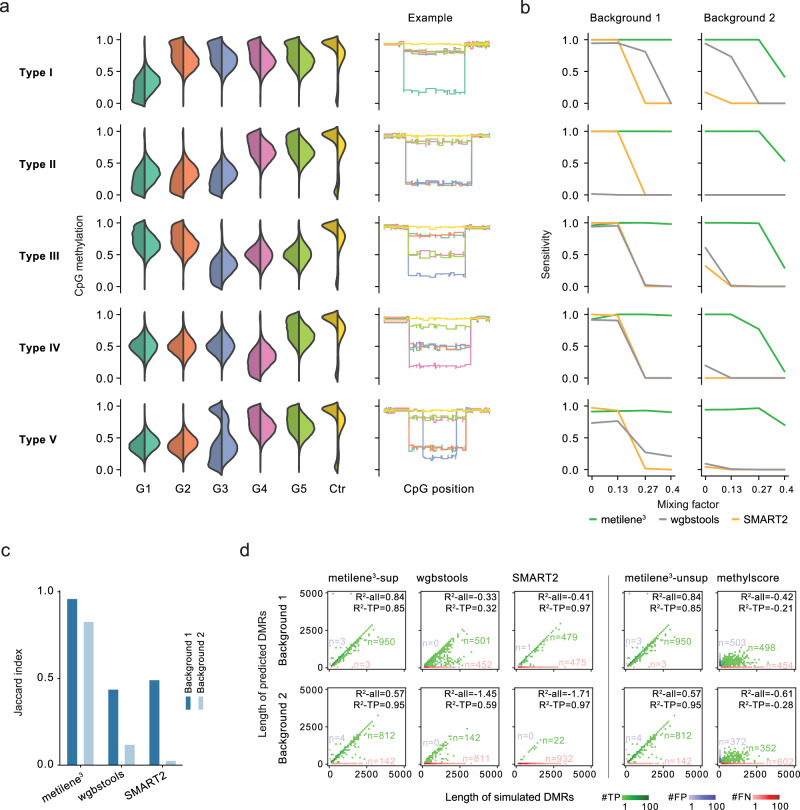
Highly accurate DMR detection using metilene^3^. **a** Methylation rate distribution and example patterns for five types of simulated DMRs (left part of the violin-plot for background 1 and right part of the violin-plot for background 2). A total of 50 samples is divided into five groups, with CpG methylation rates simulated under group-specific distributions for each DMR type. Left: Distribution of CpG methylation rates across groups for each DMR type. Right: Representative examples of group-mean CpG methylation patterns for each DMR type. **b** Sensitivity of metilene^3^, wgbstools, and SMART2 in detecting DMRs under varying conditions. **c** Overall similarity between simulated DMRs and detected DMRs (Jaccard index) of metilene^3^, wgbstools, and SMART2. **d** Length of simulated DMRs versus length of predicted DMRs under supervised detection (left, including metilene^3^-supervised mode, wgbstools, and SMART2) and unsupervised detection (right, including metilene^3^-unsupervised mode and methylscore). Simulated DMRs that was not detected or had a length of overlap with a detected DMR shorter than 100 bp were marked as FN (false negative, colored in red). Detected DMRs that has an overlap with a simulated DMR smaller than 100 bp were marked as FP (false positive, colored in blue). Other detected DMRs were marked as TP (true positive, colored in green). Source data are provided as a Source Data file.

To evaluate metilene^3^, we simulated 50 whole-genome bisulfite sequencing (WGBS) samples for human chromosome 10 (low methylation at CpG-dense regions and high methylation elsewhere), each under two different methylation backgrounds (global noise; see **Methods**). The samples were divided into five groups, and a set of simulated DMRs was “injected” into each background based on the grouping (see **Methods**). Specifically, we introduced five distinct DMR types, with 200 regions per type, totaling 1000 DMRs, and contrasted them with a control group (Ctr) without any DMRs introduced (Fig. 2a, Suppl. Data 1). DMR types I to IV represented scenarios in which groups were hypomethylated, hypermethylated, or intermediately methylated. In contrast, type V reflected a more complex pattern, where DMRs in one group varied in methylation level and length creating deviations from the core DMR (“flanking regions”, Fig. 2a). Recognizing that CpGs within a DMR are rarely fully methylated or unmethylated in biological tissue samples, we introduced heterogeneity by assigning each DMR type to one of four mixing levels, with mixing factors c ranging from 1 to 0.6. In essence, lower mixing factors reduce the mean methylation differences between groups, thereby making it harder to accurately detect DMRs. To further mimic the complexity often found in real-world biological samples, we introduced two types of batch effects (1000 batch-specific unmethylated sites), sample-specific tumor purity factors (mixture of sample and control methylation values within DMRs, varying across samples), and two confounding factors C1 and C2 (500 confounder-specific DMRs for C1 or C2, respectively, see **Methods**, Suppl. Fig. 2a–d).

We first compared metilene^3^ to other multi-group DMR callers in the supervised mode only. In total, metilene^3^ identified 1116 DMRs in background 1 and 957 DMRs in background 2. In comparison, wgbstools detected 920 and 255 DMRs, while SMART2 identified 577 and 22 DMRs in the same backgrounds (all false discovery rates ~1% or less). Notably, metilene^3^ maintained high sensitivity across all DMR types, even for regions simulated with a low mixing factor (Fig. 2b). We further measured the congruence between simulated and predicted DMRs using the Jaccard index. Metilene^3^ achieved a similarity of 0.96 in background 1 and 0.83 in background 2, while other DMR callers did not reach 0.5 in both backgrounds (Fig. 2c). We further analyzed reported and missed DMRs with respect to their length, showing a high correlation between simulated and predicted lengths for metilene^3^ and SMART2 ( > 0.8 for true positives). In contrast, wgbstools tended to underestimate the length of the DMRs (Fig. 2d, left).

We also assessed the accuracy of metilene^3^’s local clustering labels of samples (hypomethylated, hypermethylated, or intermediate) by comparing them to the simulated labels. While the overall accuracy is high (0.901/0.882 in background 1/2), we noticed variance in accuracy among the DMR types: Types I and II, i.e., types with clearer group-wise differences, were recovered with higher accuracy, while the noisier types III, IV, and V showed reduced accuracy (Suppl. Fig. 2e), likely due to the more subtle simulated differences between groups. For type V DMRs, we investigated how the group-specific length of the DMR affects the number of DMRs predicted within a single simulated DMR. As expected, metilene^3^ tended to detect more DMRs with increasing lengths of group-specific regions (“flanking region length”, Suppl. Fig. 2 f, g).

For label-free DMR identification, we benchmarked metilene^3^’s unsupervised mode with methylscore. Methylscore missed a large number of DMRs (8.0–91.6% and 18.4–98.8% percent across mixing factors and backgrounds) and the length of the predicted DMRs differed strongly from the simulated ones (avg. delta = 341 bp/363 bp in background 1/2, Fig. 2d, right, Suppl. Fig. 2 h). In contrast, metilene^3^’s unsupervised mode showed the same accuracy as the supervised mode, since DMTree inferred the groups correctly, which is likely due to the focus on strong, recurrent DMRs for clustering.

Finally, we measured the time and memory consumption of metilene^3^, wgbstools, and SMART2 using our simulated data. For supervised DMR detection, metilene^3^ needed approximately 29 min and less than 0.7 GB of memory on a single core (10 cores: 12 min and 1.26 GB of memory). In comparison, wgbstools was faster, taking about 1.6 min and using 0.48 GB of memory on a single core, and performance improved further with ten cores. SMART2 was considerably slower, requiring more than one hour to complete the same task on ten cores (Suppl. Fig. 2i). The time and memory consumption of methylscore could not be measured because the workflow required two different software versions and manual processing of intermediate files.

#### Unsupervised mode - evaluation of DMTree clustering

**Fig. 3:**
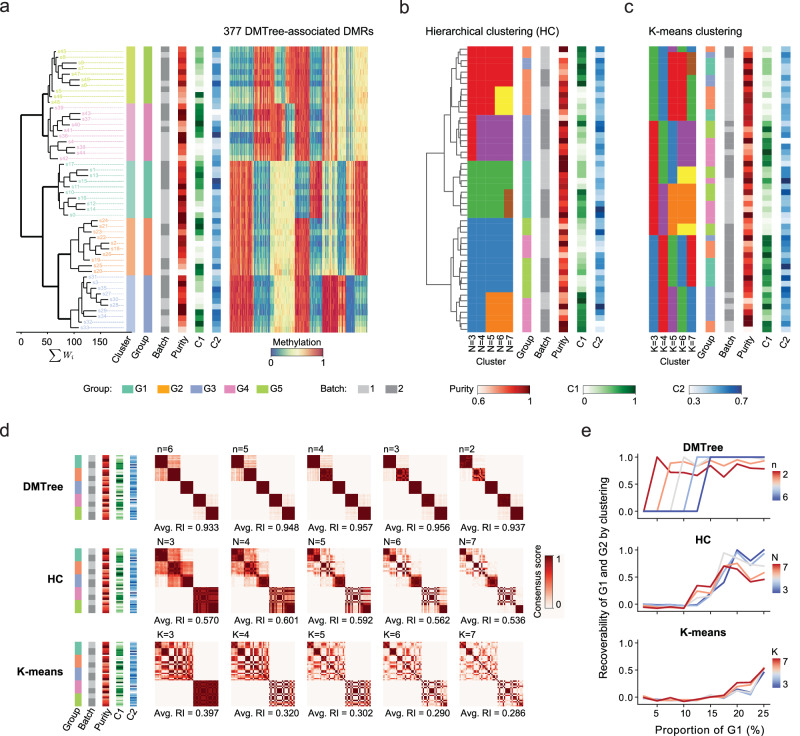
Robust and sensitive clustering with DMTree. **a** DMTree clustering (left) and corresponding heatmap (right) based on DMTree-associated unsupervised DMRs. The inferred group assignments from DMTree clustering align with the simulated group assignments across all samples. Annotation bars (middle): DMTree-based clustering, with group memberships indicated by light-colored bars, and simulated sample annotations including group, batch, purity, and confounding factors C1 and C2. The final cluster splits in the DMTree are highlighted in bold. **b** Hierarchical clustering (HC) of samples based on the top 1% most variable CpGs, with the number of clusters ranging from three to seven. **c** K-means clustering of samples based on the first two principal components of the top 1% variable CpGs, with K ranging from three to seven. **d** Consensus matrix across 50 resampled runs. Samples are ordered by simulated groups, batches, and purity. DMTree is evaluated for different minimum cluster sample sizes (n). HC and K-means are evaluated for different numbers of clusters (N for HC and K for K-means). Avg. RI: average Rand index. **e** Recoverability of groups G1 and G2 using DMTree, HC, and K-means clustering, under varying proportions of group G1 and different clustering model parameters. Recoverability is defined as the Avg. RI on samples from groups G1 and G2. Source data are provided as a Source Data file.

A central step in the unsupervised mode is assigning samples to groups based on DMRs detected across all samples (label-free) using our novel clustering method, DMTree. DMTree aggregates grouping evidence across many DMRs, so that even fine-grained but robust signals accumulate across splits, thereby improving the signal-to-noise ratio. To benchmark our algorithm against common clustering methods used for DNA methylation data, we used the previously simulated data and compared the resulting group assignments with those from hierarchical and K-means clustering (see **Methods**). In both backgrounds, DMTree accurately recapitulated the simulated groups, while hierarchical clustering and K-means clustering on the top 1% variable CpGs were affected by batch effect, especially in the noisier background 2 (Fig. 3a–c, Suppl. Fig. 3a–c).

To further challenge the robustness of the clustering methods, we performed repeated subsampling experiments (80% of the samples, 50 times, see **Methods**). To also assess the sensitivity of detecting small groups, the size of group G1 was set to a fixed number ranging from one to ten in each round (Suppl. Fig. 3 d). DMTree reported more robust clustering, achieving an average Rand index > 0.9 compared to < 0.7 for hierarchical clustering and < 0.4 for K-means clustering, and outperformed the other two methods regardless of the parameters n, N, and K of the individual algorithms (n - minimum group size in metilene^3^, N - number of groups in the hierarchical clustering, and K - number of clusters in K-means clustering, Fig. 3d). Interestingly, when utilizing those DMRs as input that were used for building the DMTree, the hierarchical and K-means clustering improved, but did not reach DMTrees levels of robustness (Suppl. Fig. 3e). Importantly, DMTree was also more sensitive to detect small groups, as it already distinguished samples from G1 and G2 (the group most similar to G1) at 5% (proportion of samples from G1), if the minimum samples in a cluster, n, was set not larger than the size of G1 (Fig. 3e). For hierarchical clustering, a proportion of G1 > 15% was required to achieve a recoverability (avg. Rand index) of G1 and G2 of 0.5, while for K-means this number was ~25%, even if a large number of clusters, K, was set (Fig. 3e).

Since real-world datasets often differ in sample purity, for example, due to blood contamination or mixed tissues, we used the TCGA cohort to capture commonly observed purity levels (median at the lowest purity level about 0.6). Based on this, we simulated four additional datasets, with decreasing levels of sample purity ranging on average from 0.8 to 0.5 (Suppl. Fig. 3 f). While the DMTree accurately predicted the sample groups for datasets where the purity was not lower than 0.6, falling below this resulted in reduced group recovery (Suppl. Fig. 3 g). Thus, commonly observed purities appeared to be well accepted, however, for extremely impure datasets the results should be taken with caution.

### DMTree recapitulates cell lineage and sample origin

To assess metilene^3^’s potential to reveal meaningful biological insights, we analyzed a large WGBS dataset of normal human cell types^13^ using the unsupervised mode. The cohort comprises 205 samples from 77 primary cell types, encompassing 39 broader categories reflecting developmental processes and tissues, e.g., samples from different stages of T cell differentiation. The resulting DMTree groups the samples into 56 clusters, showing remarkable agreement with their known cell types, functions, and tissue origins (Suppl. Fig. 4a). For instance, metilene^3^ correctly clustered pancreatic islet cell types while clearly distinguishing between alpha-, beta-, and delta cells. At the same time, metilene^3^ reported DMRs specific to each individual subtype. This included a DMR in the *CREBBP* locus hypomethylated specifically in alpha cells, and one DMR in the *ASAP1* locus hypomethylated exclusively in beta cells (Suppl. Fig. 5a).

**Fig. 4:**
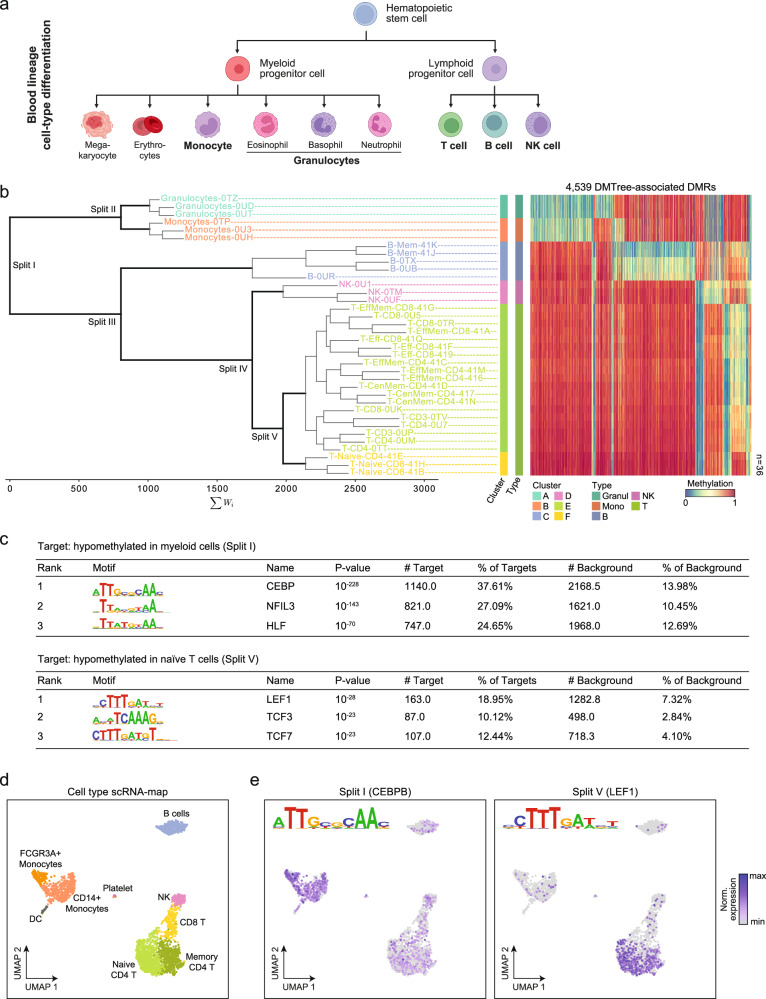
Unsupervised DMR calling with DMTree allows for precise blood lineage reconstruction based on whole-genome methylation data. **a** Schematic of blood lineage cell-type differentiation, highlighting the major splits into the myeloid and lymphoid lineage and subsequently into the subsets. Cell types present in the data are indicated in bold. Created in BioRender. Kretzmer, H. (2026) https://BioRender.com/q8djq5y. **b** DMTree clustering (left) and corresponding heatmap (right) based on DMTree-associated unsupervised DMRs in 36 WGBS blood samples (parameters: minimum samples per cluster: 3, minimum absolute DMR difference: 0.5, minimum total methylation difference ${{\rm{W}}}$: 100). The first split distinguishes myeloid from lymphoid lineages. Subsequent splits separate Granulocytes from Monocytes and B cells from Natural Killer (NK) cells and T cells, respectively. Within the lymphoid lineage, NK cells are further separated from T cells. The final split, not explicitly defined in the reference, differentiates naïve from more mature T cells. Notably, the clusters derived from unsupervised DMR calling align exactly with the annotated cell types. **c** The top three enriched motifs in DMRs associated with split I (top, separating myeloid from lymphoid lineages) and split V (bottom, distinguishing naïve T cells from more mature T cells). **d** Published scRNA-seq data for immune cell types corresponding to those identified in the unsupervised DMR analysis. The dataset includes Monocytes, NK cells, B cells, and T cells, with each cell type color-coded by its annotation. Data are visualized using Uniform Manifold Approximation and Projection (UMAP) to illustrate cell-type clustering. **e** Expression of transcription factors (TFs) identified from scRNA-seq data. The shown TFs are associated with motifs enriched in DMRs corresponding to specific DMTree cluster splits (see **b**). Top-left insets show the enriched motifs for each TF, while cells are color-coded by normalized expression levels. Left: CEBPB shows higher expression in Monocytes than in the lymphoid lineage. Right: LEF1 shows a distinct naïve T cell-specific expression pattern. Source data are provided as a Source Data file.

While the atlas spans a broad range of tissues, most studies focus on specific cell types or organs. To assess metilene^3^’s ability to differentiate between highly similar cell types, we also applied it to 36 blood samples annotated as five distinct cell types, covering various differentiation stages (Fig. 4a). This analysis revealed 6 clusters and identified 363,861 DMRs (Suppl. Data 2). The resulting DMTree recapitulated key lineage relationships, with five major splits: first separating myeloid cells from lymphocytes, then granulocytes from monocytes, followed by B cells from other lymphocytes, NK cells from T cells, and finally naïve from mature T cells (the latter were not distinguished in the original analysis of the atlas, Fig. 4b). Notably, when using methylation levels from metilene^3^-derived DMRs, samples grouped clearly in a PCA by cell type, whereas no clear separation was observed when using all CpGs, the top 1% variable CpGs, nor the same number of CpGs as used for DMTree (Suppl. Fig. 5b). This highlights the cell type specificity of the identified DMRs and prompts the question whether these DMRs could have functional relevance in the differentiation of the hematopoietic lineage.

To assess the functional relevance of the DMRs identified by metilene^3^, we investigated whether potential transcription factor binding sites within these regions are associated with blood cell-specific functions. To this end, we performed a motif enrichment within DMRs linked to DMTree splits. Notably, we identified a significant enrichment of the ATTGCGCAAC motif in split I (hypomethylated in myeloid cells), which is recognized by C/EBPβ, and the CCTTTGATST motif in split V (hypomethylated in naïve T cells), resembling the DNA binding motif of Lymphoid enhancer-binding factor 1 (*LEF1*) (Fig. 4c). To evaluate whether these transcription factors are also associated with cell-type-specific expression patterns, we analyzed a published single-cell RNA-seq dataset from human peripheral blood mononuclear cells (Fig. 4d). The dataset comprises lymphocytes (T, B, and NK cells) and monocytes but does not include granulocytes. Consistent with previous studies^19^, C/EBPb is highly expressed in monocytes compared to lymphocytes, supporting the notion that DMRs driving the first major DMTree split reflect a biologically relevant lineage separation (Fig. 4e). Notably, C/EBPb has been shown to induce the transdifferentiation of v-ABL-immortalized primary B cells, which generally belong to the lymphocytic lineage, into granulocyte-macrophage progenitor-like cells (GMP-like cells), characteristic of the myeloid lineage^20,21^. Further down the lineage, LEF1 plays a crucial role in T cell development by maintaining genomic organization and ensuring the stability of their identity and function^22^. Indeed, *LEF1* expression is highest in naïve CD4^+^ T cells, as observed previously^23^ (Fig. 4e). These observations highlight that metilene^3^-identified DMRs capture relevant regulatory regions involved in shaping cell identity and thereby offer insights into underlying gene regulatory networks.

### Application to cancer datasets

Cancer samples are typically more heterogeneous than healthy tissues, rendering the detection of DMRs considerably more challenging. To assess whether metilene^3^ can be utilized to biological hypotheses in such complex contexts, we applied metilene^3^ to two publicly available cancer-related WGBS datasets^24,25^ as well as a dataset derived from cell-free DNA^26^.

#### Glioblastoma

**Fig. 5:**
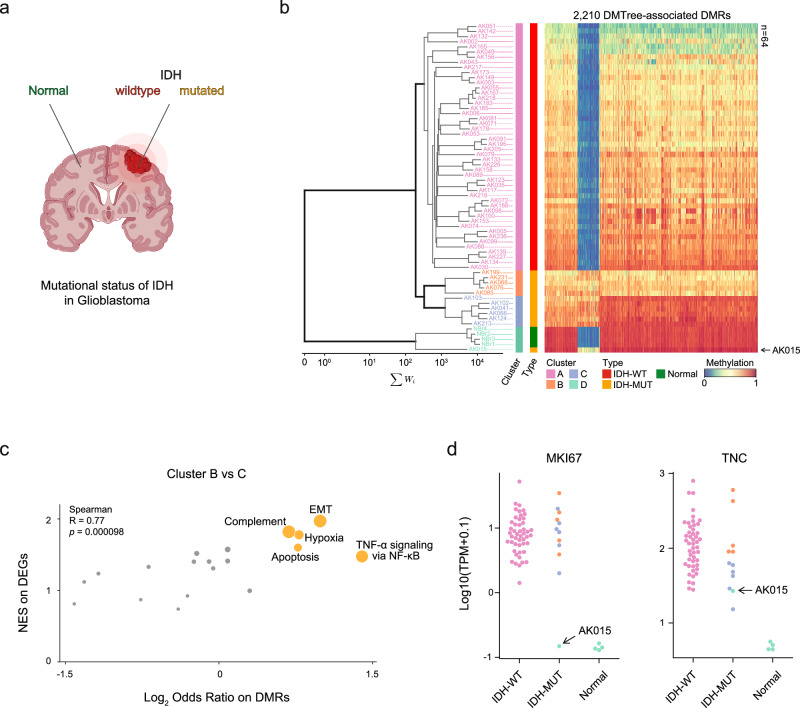
Precise classification in brain tumors with DMTree. **a** Schematic of a brain with an indicated tumor in red. The following analysis focuses on IDH-mutated and unmutated Glioblastoma and normal controls. Created in BioRender. Kretzmer, H. (2026) https://BioRender.com/5ddax3s. **b** DMTree clustering (left) and corresponding heatmap (right) based on DMTree-associated unsupervised DMRs in 64 WGBS samples from IDH wildtype (WT) and mutated (MUT) Glioblastoma, as well as normal controls (parameters: minimum samples per cluster: 4, minimum absolute DMR difference: 0.5, minimum total methylation difference ${{\rm{W}}}$: 100). The first split distinguishes Glioblastoma samples from normal controls, except for a single IDH-MUT Glioblastoma sample (AK015), which clusters with normal controls. Subsequent splits separate IDH-MUT from IDH-WT samples, and further classify two phenotypically uncharacterized subgroups within the IDH-MUT samples (Clusters B and C). **c** Gene set enrichment analysis (GSEA) comparing differentially expressed genes (DEGs) and differentially methylated regions (DMRs) between IDH-MUT subclusters B and C. Normalized enrichment scores (y-axis) are plotted against odds ratios (x-axis) for hallmark gene sets (Suppl. Data 5). The dot size corresponds to the combined *p*-value (Fisher’s method) from GSEA analysis on DEGs and DMRs, with smaller *p*-values represented by larger dots. Gene sets with a combined *p*-value < 0.01 are colored. The most prominent differences between the two IDH-MUT subgroups are observed in pathways associated with epithelial-to-mesenchymal transition (EMT), complement, hypoxia, apoptosis, and TNF-α signaling via NF-κB. Two-sided Spearman’s correlation coefficient with associated *p*-value is calculated. **d** Expression levels (TPM) of MKI67 and TNC, genes associated with proliferation and tumor plasticity in Glioblastoma. The Glioblastoma sample AK015, which clusters with normal control samples in (**b**), exhibits normal MKI67 expression levels but shows high expression of TNC, indicating that AK015 is indeed a tumor sample with expression levels comparable to other IDH-MUT and IDH-WT Glioblastomas. Source data are provided as a Source Data file.

The dataset from Wu et al.^24^ includes 64 samples, from a mixed cohort of IDH-mutated and IDH-wild-type glioblastoma tumors, as well as non-cancerous brain tissues (Fig. 5a). The unsupervised DMTree identified 267,811 DMRs and partitioned the samples into four groups (Fig. 5b, Suppl. Data 3). Cluster A contained IDH-wildtype tumors, clusters B and C comprised IDH-mutated tumors, and cluster D is primarily composed of non-cancerous samples, demonstrating correct stratification by pathobiology and IDH mutation status (Fig. 5b). To assess whether the IDH mutation-associated clusters B and C represent biologically distinct subgroups, we calculated differentially expressed genes (DEGs) and performed gene-set enrichment analyses (GSEA) on both DMRs and DEGs (Suppl. Data 4 and 5). The GSEA suggested potential molecular subtypes that are differentially enriched for cancer-associated pathways, such as cluster B being associated with genes involved in epithelial-mesenchymal transition (EMT). Notably, the DMR-based odds ratios significantly correlated with the GSEA’s network enrichment scores calculated from DEGs (Spearman’s *R* = 0.77, *p* < 0.0001), indicating concordant changes in methylation and gene expression (Fig. 5c).

Motif analysis in these DMRs suggests that a Krüppel C2H2 zinc-finger TF (ZNF766, *p* < 10^-27^) may be involved in the methylation differences between the detected clusters B and C (Suppl. Fig. 6c). ZNF766 is broadly expressed in healthy brain and other tissues^27^ and has recently been implicated in the interference with PRDM9-mediated recombination^28^ and thus may be linked to chromosomal instability^29,30^. Interestingly, another enriched TF, CTCFL (BORIS), which is typically only expressed during spermatogenesis, was found to be activated by PRDM9^28,31^. We also observed significant enrichment of CDX1 (*p* < 10^−27^), a transcription factor associated with proliferation and apoptosis. Among other TFs, our data indicate binding of ZNF766, CTCFL, and CDX1 in the B-cluster of IDH-mutated glioblastomas, suggesting potential regulatory differences to the remaining IDH-mutated glioblastomas in cluster C.

Furthermore, we identified an apparent IDH-mutated tumor outlier, AK015, that clustered with normal samples in DMTree. This sample also showed atypical transcriptomic features: expression of the proliferation marker *MKI67* was as low as in normal samples, suggesting reduced proliferative activity compared to other tumors (Fig. 5d). However, *TNC*, a glioma-associated antigen, remained highly expressed, arguing against a simple tumor-purity explanation and supporting its tumor identity (Fig. 5d). In addition, specifically for the IDH-mutant-specific DMRs, AK015 deviated from the other normal samples and showed a partial shift towards cancer, whereas other cancer-specific regions appeared more normal-like. (Fig. 5b). PCAs based on all, top-variable or DMR-CpGs, as well as on the transcriptomic data, corroborated this intermediate profile (Suppl. Fig. 6a, b). While elucidating the precise cause of the sample’s atypical signature is beyond the scope of this paper, metilene^3^ highlighted the sample as an outlier, enabling further research into the underlying reason.

Finally, we tested how sensitive metilene^3^ is when applied to more challenging samples due to potentially lower sample purity. To this end, we utilized a brain cancer dataset^26^ of circulating cell-free DNA (cfDNA) from cerebrospinal fluid (CSF), i.e., small DNA fragments released into the CSF, often originating from brain tumors or nearby tissues. We tested metilene^3^ on nine cfDNA samples, four from non-tumor donors (hydrocephalus patients) and five from different treatment stages of medulloblastoma patients, which the DMTree successfully clustered into tumor and non-tumor groups (Suppl. Fig. 7, Suppl. Data 6).

#### Pancreatic ductal adenocarcinoma

**Fig. 6:**
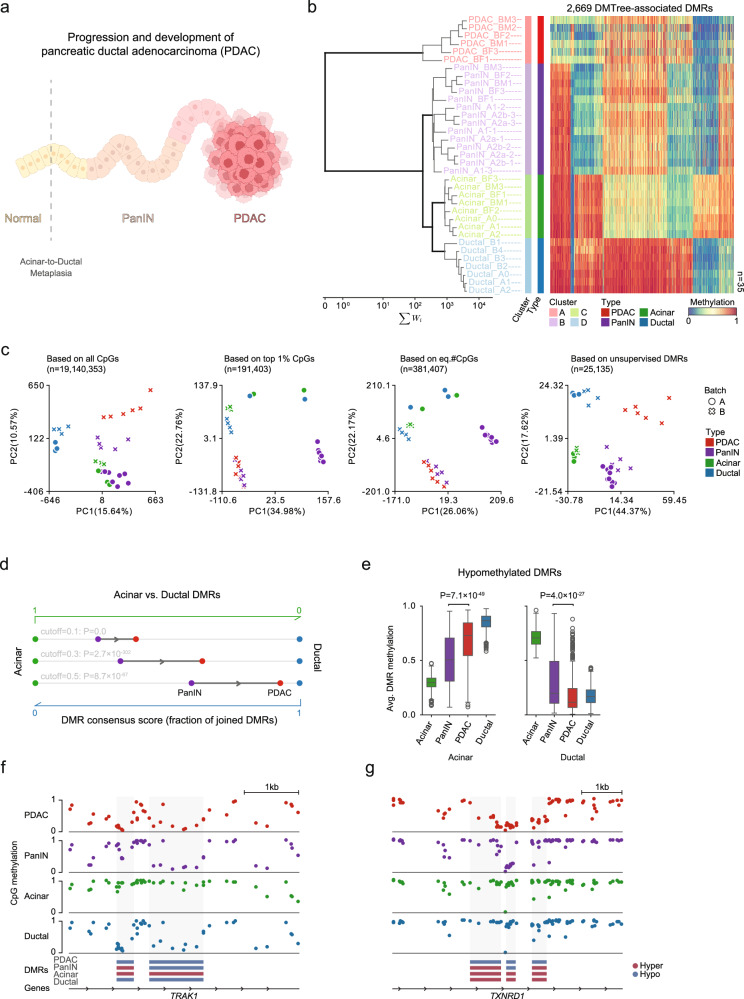
Metilene^3^ captures the acinar-to-ductal transformation and hints towards the cell-of-origin in PDAC. **a** Schematic of the progression and development of pancreatic ductal adenocarcinoma (PDAC) from normal acinar or ductal cells through the pre-cancerous lesion tissues (PanIN). Created in BioRender. Kretzmer, H. (2026) https://BioRender.com/part5nh. **b** DMTree clustering (left) and corresponding heatmap (right) based on DMTree-associated unsupervised DMRs in 35 WGBS samples from PDAC, PanIN, and normal acinar and ductal tissues (parameters: minimum samples per cluster: 4, minimum absolute DMR difference: 0.5, minimum total methylation difference ${{\rm{W}}}$: 100). The first split separates PDAC from non-cancerous tissues. Non-cancerous samples are subsequently subdivided into pre-cancerous lesions (PanIN) and benign tissues, which are further classified by cell type (acinar vs. ductal). The inferred clusters align precisely with the annotated sample conditions. **c** PCA of CpG methylation in pancreas tissue samples. (Left) PCA based on all CpGs primarily separates ductal cells (PC1) from acinar, PanIN, and PDAC samples, with minimal distinction between acinar and PanIN tissues. (Middle left and middle right) PCA based on the most variable CpGs (middle left: top 1%, middle right: equivalent number of CpGs used to build DMTree) shows batch-effect associated separation (A: Swift Accel-NGS Methyl-Seq, B: NEBNext Ultra DNA). (Right) PCA based on unsupervised DMRs achieves a clear separation of all four cell types, with PC1 distinguishing normal tissues from pre-cancerous (PanIN) and malignant (PDAC) samples, while PC2 separates the two normal control cell types (ductal and acinar) as well as the pre-malignant and malignant tissues. **d** DMR consensus scores indicating methylation similarity between groups. The consensus score reflects the fraction of DMRs supporting the clustering of PanIN or PDAC with acinar or ductal, respectively. PanIN exhibit greater methylation similarity to acinar tissues, whereas PDAC appears more ductal-like. Lines indicate Δ-methylation thresholds (0.1, 0.3, 0.5) for DMRs (*n* = 163,775, 14,165, 1,804) considered in the consensus calculation. Dots on the left represent samples clustering more frequently with acinar, while dots on the right indicate a stronger association with ductal tissue. *P*-value are calculated with two-sided Fisher’s exact test. **e** Distribution of mean DMR methylation across groups and DMR types. Left: DMRs hypomethylated in acinar tissue and hypermethylated in ductal tissue exhibit lower methylation levels in PanIN than in PDAC (*n* = 820 DMRs). Right: DMRs hypomethylated in ductal tissue and hypermethylated in acinar tissue display lower methylation levels in PDAC compared to PanIN (*n* = 674 DMRs). Both comparisons reinforce the higher similarity of PanIN with acinar tissue, while PDAC samples appear further along the acinar-to-ductal transformation, resembling ductal samples more closely. Two-sided Wilcoxon rank-sum test is applied. Boxes show the first and third quartiles with the median at the centre and whiskers extend to the minimum and maximum within 1.5x the inter-quartile range. **f** CpG methylation patterns at two DMRs covering the TRAK1 gene (chr3:42,189,735-42,193,918). Acinar tissue exhibits high methylation across both DMRs, whereas ductal and PDAC show strong hypomethylation. PanIN display a mixed pattern, with the first DMR remaining highly methylated, while the second, longer DMR is predominantly hypomethylated. DMRs are highlighted in grey, with methylation status indicated in blue (hypomethylation) and red (hypermethylation). **g** CpG methylation patterns at three DMRs covering the TXNRD1 gene (chr12:104,663,406-104,669,010). All three DMRs distinguish hypomethylated PDAC samples from the highly methylated benign tissues. PanIN samples exhibit an intermediate methylation pattern, where the middle DMR begins to show hypomethylation similar to PDAC, while the flanking DMRs remain hypermethylated, distinguishing PDAC from PanIN and benign tissues. DMRs are highlighted in grey, with methylation status indicated in blue (hypomethylation) and red (hypermethylation). Source data are provided as a Source Data file.

We re-analyzed data of Lo et al.^25^, comprising 35 samples of acinar and ductal cells, pancreatic intraepithelial neoplasia (PanIN), and pancreatic ductal adenocarcinoma (PDAC) (Fig. 6a). Metilene^3^ identified 275,352 DMRs and constructed a DMTree that accurately separates cancer from non-cancer samples, neoplastic from normal samples, and acinar from ductal samples (Fig. 6b, Suppl. Data 7). A PCA based on DMR methylation confirmed separation of neoplastic and both normal types (Fig. 6c, right). In contrast, a PCA using all or the top-variable CpGs did not show a straightforward separation between acinar and neoplastic samples and instead captured a potential library-preparation batch effect (samples names containing A - Swift Accel-NGS Methyl-Seq and B - NEBNext Ultra DNA, Fig. 6c).

Given ongoing debates regarding the cell-of-origin, acinar or ductal, from which individual PDACs may arise^32^, we computed consensus scores reflecting methylation-based similarities of neoplasia and PDAC samples to acinar or ductal samples using acinar-ductal DMRs (see **Methods**). In line with the acinar-to-ductal metaplasia (ADM) model^32^, PanIN samples were consistently more similar to acinar cells, while PDAC samples more closely resembled ductal cells (Fig. 6d), supporting the idea that PanINs represent an intermediate state in the transition from acinar to ductal identity during tumor progression. Notably, when restricting to DMRs with larger acinar-ductal differences, the similarity of PanIN and PDAC samples to ductal cells increased. This suggests that PanINs acquire ductal-like methylation signatures in regions where normal acinar and ductal cells differ most strongly, highlighting their transitional status in PDAC development. Further stratification of acinar-ductal DMRs showed that regions hypomethylated in acinar cells gained methylation gradually, whereas those hypomethylated in ductal cells became demethylated more abruptly during progression (Fig. 6e). Across all analyses, PanIN methylation consistently fell between acinar and PDAC, supporting an acinar-PanIN-PDAC trajectory within the acinar-to-ductal metaplasia (ADM)^33^ framework.

Next, we examined the composition and genomic distribution of the DMRs. Metilene^3^ detected closely spaced DMRs with different behavior across groups, which is a direct benefit of its local clustering strategy. For example, a DMR downstream of the *TRAK1* transcription start site was hypomethylated in PDAC and ductal samples, but not in PanIN and acinar samples, whereas an adjacent DMR was exclusively hypermethylated in acinar cells (Fig. 6f). A similar pattern was observed in an intron of *TXNRD1*: three DMRs showed hypomethylation in PDAC, while both normal tissues were hypermethylated (Fig. 6g). In neoplastic samples, one of the DMRs was also hypomethylated, whereas the others retained normal-like methylation. At both loci, these epigenetic changes were accompanied by a gradual upregulation of expression, consistent with ADM (Suppl. Fig. 8a). *TRAK1*, which encodes a mitochondrial trafficking kinesin, has been linked to invasive behavior of other cancers^34^, and may be relevant to the invasive nature of PDAC^35^. Similarly, *TXNRD1* encodes thioredoxin reductase 1, a cancer-associated enzyme and FDA-approved therapeutic target involved in antioxidant defense in pancreatic β-cells^36^, and its overexpression is associated with poor prognosis in hepatocellular carcinoma via AKT/mTOR activation^37^. Although neither gene has been directly linked to PDAC, their epigenetic double-switch patterns suggest potential roles in disease mechanisms and warrant further investigation.

To characterize the first DMTree split, we searched for transcription factor binding sites potentially involved or affected by the differential methylation in PDAC samples (Fig. 6b). Motif enrichment analyses on PDAC-DMRs showed no significant enrichment among strongly PDAC-hypermethylated regions (mean methylation loss > 0.5, *n* = 125). In contrast, several motifs were significantly enriched in strongly PDAC-hypomethylated DMRs (mean methylation loss > 0.5, *n* = 524, Suppl. Fig. 9a). Using all remaining DMRs (*n* = 8661) as background, we observed strong enrichment of Nuclear factor-κB 2 (NFKB2; OR = 3.35, *p* = 8.3 × 10^−23^) and Nuclear factor of activated T-cells, cytoplasmic 1 (NFATc1; OR = 2.26, *p* = 2.7 × 10^−11^) motifs in PDAC-hypo DMRs (Suppl. Fig. 9b). NF-κB signaling is known to promote carcinogenesis by inhibiting apoptosis and fostering proliferation, angiogenesis, and metastasis^38^, and NFAT factors (NFATc1–4) regulate cell cycle, survival, migration, and angiogenesis, and have been implicated in PDAC^39,40^. Notably, 34% of PDAC-hypo DMRs (179/524) contained either an NFKB2 or an NFATc1 motif, and 8% (44/524) harbored both motifs, representing a significant enrichment over background (OR: 9.83; Fisher’s exact test *p*-value = 3.8 × 10^−24^, Suppl. Fig. 9b, Suppl. Data 8). Genome-wide, only 1.6% of the NFATC1 motifs occur within 30 bp of an NFKB2 motif, compared to 38.7% in PDAC-hypo DMRs. Notably, both binding sites are members of an extended set of transcription factor binding sites showing a pronounced co-occurrence, which cannot be attributed to DMR length, as PDAC-hypo DMRs were, on average, shorter than other DMRs (Suppl. Fig. 9c, d).

**Fig. 7:**
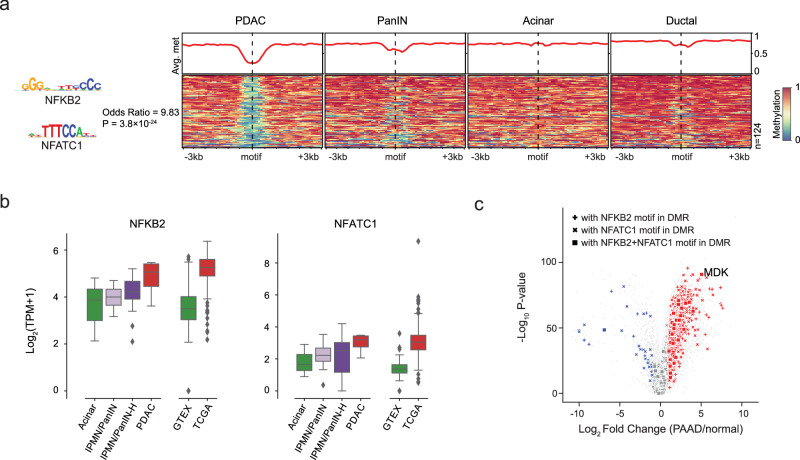
Metilene^3^ reveals co-binding of NFKB2 and NFATC1 in PDAC. **a** Smoothed methylation of DMRs with both NFKB2 motif (VGGGRATTYCCC) and NFATC1 motifs (ATTTTCCATT), centered on the motif sites. PDAC exhibits strong unmethylation at these motifs; PanIN shows intermediate methylation; and normal tissues show predominantly methylated patterns. The co-binding of NFKB2 and NFATC1 was significantly enriched in DMRs that were hypomethylated in PDAC (Fisher’s exact test, two-sided). **b** Expression of NFKB2 and NFATC1 across acinar (*n* = 3), IPMN/PanIN (*n* = 18), high-grade IPMN/PanIN (IPMN/PanIN-H, *n* = 16), PDAC (*n* = 4), GTEx-pancreas (*n* = 167), and TCGA’s pancreas adenocarcinoma (PAAD, *n* = 179), from GSE210351^42^ and UCSC Xena^43^. Both genes show a progressive increase in expression from normal tissues to cancer. IPMN: intraductal papillary mucinous neoplasm. PanIN: pancreatic intraepithelial neoplasia. Boxes show the first and third quartiles with the median at the centre and whiskers extend to the minimum and maximum within 1.5x the inter-quartile range. **c** Differentially expressed genes (DEGs) from the comparison of TCGA’s pancreas adenocarcinoma and the normal tissues, from GEPIA2. Four-way analysis of variance (ANOVA) is applied. Genes are classified based on whether their associated DMRs contain NFKB2 or NFATC1 binding motifs. MDK is the most significant DEG that contains a DMR with an NFKB2 motif and an NFATC1 motif. Source data are provided as a Source Data file.

We next asked whether their TFBSs are more generally associated with differential methylation. Across all DMRs (not only PDAC-hypo), we observed symmetric hypomethylation centered on NFKB2 and NFATc1 motifs (Suppl. Fig. 9e, left). Importantly, Arlt et al. identified NF-κB and NFAT (with Nrf2) as major nuclear factors contributing to PDAC development^41^ and hypomethylation of the NFKB or NFAT motifs was strongest in PDAC and, to a lesser extent, also present in PanIN (markedly weaker in acinar and ductal samples). Furthermore, DMRs harboring these motifs lie near genes enriched for WNT/β-catenin and TNFα signaling via NF-κB pathways (Suppl. Fig. 9e, right). In dual-motif DMRs, PanIN methylation resembled almost normal tissue (Fig. 7a), indicating that dual-motif hypomethylation is more PDAC-specific than single-motif sites. Consistent with this, public RNA-seq data^42,43^ show gradual upregulation of NFKB2 and NFATC1 (and family members) along the ADM continuum, peaking in PDAC (Fig. 7b; Suppl. Fig. 10a). Integrating metilene^3^-detected DMRs with DEGs between TCGA-PAAD samples and matched normal tissues from GEPIA2^44^, we identified the midkine gene (MDK) as the most prominently upregulated DEG associated with a dual-motif DMR (Fig. 7c and Suppl. Fig. 10b, c, Suppl. Data 9). Prior work implicated MDK in promoting EMT^45^, enrichment in extracellular vesicles of pancreatic cancer cells^46^ and increased proliferation and migration of pancreatic cancers^47^.

Together, these data support a hypothesis-generating model in which PDAC-specific dual-motif hypomethylation marks regulatory elements near MDK and other cancer-progression associated genes, with possible coupling to NF-κB/NFAT activity (potentially via Akt/mTORC1/NF-κB^45^; Suppl. Fig. 10 d). Beyond MDK, metilene^3^ highlights loci with complex methylation patterns (e.g., *TRAK1*, *TXNRD1*) as candidates for biomarker development and mechanistic follow-up (Suppl. Data 9).

## Discussion

Here, we proposed metilene^3^ to analyze whole-genome methylation data, enabling multi-condition unsupervised DMR identification and the construction of a DMR-based tree (DMTree) for sample clustering. Building on an improved circular binary segmentation algorithm, metilene^3^ achieves high performance in simulations (sensitivity 96.8%/83.2% and precision 98.9%/99.0% in background 1/2). Furthermore, the DMTree accurately clustered all simulated samples as well as samples from normal human tissues and tumors. By coupling label-free DMR detection with DMR-anchored clustering, methylation analysis becomes less dependent on prior knowledge and less biased. Importantly, DMTree provides direct interpretability, as individual DMRs are explicitly linked to tree splits, making clustering results more intuitive, interpretable, and verifiable. Finally, metilene^3^ is fast and memory-efficient: using 10 cores, a human cancer WGBS dataset of 35 samples was processed in ~10 min with less than 1 GB of memory, enabling parameter tuning for exploratory data analysis.

We demonstrated that applying metilene^3^ to biomedical datasets holds the potential to provide critical insights into genome regulatory interactions. The DMTree recapitulated blood cell lineage relationships and re-identified cell-type-specific regulatory regions, factors, and targets in a human cell atlas^13^. Likewise, in more challenging settings, such as cancer and cfDNA datasets, metilene^3^ identified plausible DMRs and DMTrees, enabling exploratory analyses and biological hypothesis development. Of note, as with any clustering approach, continuous trajectories are represented as distinct branches; however, underlying similarities are preserved in the tree topology, and future extensions could incorporate diffusion-based methods to explicitly model continuous data. In pancreatic tissues, metilene^3^ clearly distinguished pancreatic adenocarcinoma, neoplastic, and control tissue samples. Among these, metilene^3^ reported highly discriminative, cancer-associated DMRs enriched for motifs suggesting NFKB and NFAT co-binding. The integration of RNA expression data makes it tempting to speculate on a potential midkine–NF-κB–NFAT signaling axis involved in pancreatic tumorigenesis. Beyond pathway-level signals, metilene^3^ also highlights individual loci with complex methylation patterns that differentiate healthy, neoplastic, and malignant tissues. These loci represent promising targets for mechanistic follow-up and potential diagnostic biomarkers.

Future work will focus on extending the application scope of metilene^3^ to other technologies. Due to the challenge of acquiring high-coverage Oxford Nanopore Technologies or PacBio direct DNA sequencing data, we did not specifically test this scenario. However, several studies have shown that the methylation rates between these technologies are comparable. Therefore, we speculate that no significant bias is expected. As single-cell bisulfite sequencing technologies (scBS) improve, metilene^3^ is prepared to handle scBS due to its standardized data input format and ability to handle large sample sizes; however, to date, the high dropout rate in this technology poses a significant challenge for prior data imputation.

In conclusion, metilene^3^ offers a sensitive, accurate, and efficient solution for analyzing methylation data with limited prior knowledge and has the potential to enhance the development of new hypotheses and discoveries in biomedical studies, to be further tested in experimental or translational settings.

## Methods

### Segmentation

We modified the circular binary segmentation described in Jühling et al.^5^. Here, we first introduce our basic approach to segmentation and subsequently explain how this is done in the unsupervised mode.

### Basic pair-wise segmentation strategy

Let $E$ denote the set of samples. For some group of samples, i.e., $G\subset E$, we calculate the mean methylation level at the cytosine $i$ with

1 $${M}_{i}\left(G\right)=\frac{1}{\left|G\right|} \mathop{\sum }\limits_{e\in G}{\beta }_{i,e}$$

where ${\beta }_{i,e}$ is methylation value of CpG $i$ in the sample $s$. Consequently, the mean difference signal (MDS), $\varDelta$, for cytosine $i$ between two groups $G,H\subset E$ with $G\cap H={{\varnothing }}$ is calculated by

2 $${\Delta }_{i}\left(G,H\right)={M}_{i}\left(G\right)-{M}_{i}\left(H\right)$$

and the sum of such differences over sum interval $\left[u,v\right]$ is

3 $${S}_{u,v}\left(G,H\right)=\mathop{\sum }\limits_{i=u}^{v}{\varDelta }_{i}\left(G,H\right)=\mathop{\sum }\limits_{i=0}^{v}{\varDelta }_{i}\left(G,H\right)-\mathop{\sum }\limits_{j=0}^{u-1}{\varDelta }_{j}\left(G,H\right)$$

Subsequently, the MDS in some interval $\left[s,t\right]$ is segemented using a Z-score. Specifically, the algorithm determines the coordinates $\left[u,v\right],s\le u < v\le t$, maximizing the score for two given groups $G,H$ with

4 $$\mathop{{{\rm{argmax}}}}\limits_{s\le u < v\le t}\,{Z}_{s,t}\left(u,v{{\rm{| }}}G,H\right)$$

where

5 $${Z}_{s,t}\left(u,v{{\rm{| }}}G,H\right)=\frac{{\left[\left|{S}_{u,v}\left(G,H\right)\right|-\frac{v-u}{t-s}\cdot \left|{S}_{s,t}\left(G,H\right)\right|\right]}^{2}}{\left(v-u\right)\left[1-\frac{v-u}{t-s}\right]}$$

The segmentation algorithm proceeds recursively until no further increase of a two-dimensional Kolmogorov-Smirnov (KS) test statistic is achieved. To be computationally efficient, MDS with ${\sum }_{i=u}^{v}\max \left[0,{\mathrm{sgn}}\left(\left|{\Delta }_{i}\left(G,H\right)\right|-\delta \right)\right] < {r}_{\min }$, where $\delta$ is a user-defined methylation difference threshold (default: 0.5) and ${r}_{\min }$ is a user-defined minimum CpG with difference exceeding $\delta$ (default:5), will be omitted and no 2D-KS test will be performed. Once a segment is found that satisfies the minimal methylation difference $\delta$ and has no subintervals with better test statstics, it is reported as DMR.

### Segmentation in the unsupervised mode

In the unsupervised mode, for some interval $\left[s,t\right]$, metilene^3^ assigns the two samples maximizing the Z-score in $\left[s,t\right]$, $O$ and $R$, to groups ${G}^{{\prime} }$ and ${H}^{{\prime} }$, respectively. After determining the Z-score maximizing interval $\left[u,v\right]$, metilene^3^ proceeds by clustering the remaining samples in $E\setminus \{{G}^{{\prime} }\cup {H}^{{\prime} }\}$. The cluster index $C$ assigning the samples to one or none of the two groups is calculated by

6 $${C}_{u,v}\left(e\right)={sgn}\left({d}_{u,v}\left(e,{G}^{{\prime} }\right)\right)-{sgn}\left({d}_{u,v}\left(e,{H}^{{\prime} }\right)\right)$$

using the pseudo-distance function

7 $${d}_{u,v}\left(A,B\right)=\frac{{\sum }_{i=u}^{v}{max} \left[0,{sgn}\left(\left|{\Delta }_{i}\left(A,B\right)\right|-{{\rm{\varepsilon }}}\right)\right]}{\left(v-u\right)}-\,\rho$$

where $\epsilon$ is a user-defined methylation threshold (default: 0.5) and $\rho$ (default:0.5) is a user-defined bias. In brief, the function $d$ sums up the number of cytosines with an absolute mean difference between $A$, e.g., the sample $e$, and $B$, e.g., the set $G$, that is strictly larger than $\epsilon$. This number is normalized to the size of the interval. The bias term $\rho \ge 0$ shifts this value, making it harder to achieve positive distances. In turn, for the calcuation of the cluster index $C$, higher values of $\rho$ shrink the decision boundary, makeing the classification more conservative and assigning more samples to none of the two groups (${C}_{u,v}=0$). Consequently, for ${C}_{u,v}\left(e\right) < 0$, $e$ will be added to a new group $G$, for ${C}_{u,v}\left(e\right) > 0$, $e$ will be added to a new group $H$. For ${C}_{u,v}\left(e\right)=0$, samples are assigned to the intermediate group $I$. Within the segment $\left[u,v\right]$, the two-dimensional KS (2D-KS) test for equality is applied to $G$ and $H$. The determined groups also play a critical role for the DMTree explained below.

### Supervised multi-group segmentation

In the supervised multi-group segmentation mode, metilene^3^ performs the steps outlined in the basic segmentation strategy, i.e., the Z-score calculations for all possible combinations of groups. Once the pair of groups ${G}^{{\prime} }$ and ${H}^{{\prime} }$, as well as the region $\left[u,v\right]$, that maximize the Z-score are found, metilene^3^ clusters the remaining groups with the group-version cluster index ${C}_{u,v}\left(T\right)$:

8 $${C}_{u,v}\left(T\right)={sgn}\left({d}_{u,v}\left(T,{G}^{{\prime} }\right)\right)-{sgn}\left({d}_{u,v}\left(T,{H}^{{\prime} }\right)\right)$$

Three new groups will be first initialized: $G\leftarrow {G}^{{\prime} }$, $H\leftarrow {H}^{{\prime} }$, $I\leftarrow {{\varnothing }}$. For ${C}_{u,v}\left(T\right) < 0$, $T$ will be merged with the group $G$. For ${C}_{u,v}\left(T\right) > 0$, $T$ will be merged with the group $H$. For ${C}_{u,v}\left(T\right)=0$, $T$ will be merged with the group $I$. The new groups $G$ and $H$ will be used for 2D-KS test within $\left[u,v\right]$.

In addition to the 2D-KS test, Kruskal–Wallis test on the mean methylation level of DMRs will be performed across all groups using scipy^48^, with a default *p*-value threshold 0.01.

### DMTree construction

We recall that metilene^3^ has locally assigned samples to one of three sets during each recursion step of the unsupervised segmentation. Thus, at the end of the segmentation, for some DMR $k$, there is set ${G}_{k}$, ${H}_{k}$ and ${I}_{k}$. In the following, we will use these local assignments to build the differential methylation tree (DMTree) by performing recursive binary splits. In the following we assume that some set ${{\bf{K}}}$ holds all DMRs. For the DMR $k\in K$, the set ${G}_{k}$ holds the hypo-methylated samples and the set ${H}_{k}$ holds the hyper-methylated samples. Also, let $\sigma :\{1,\ldots,\left|E\right|\}\to E$ be an indexing function that returns the $i$-th element of the sample set $E$.

The algorithm recursively splits the set of samples into smaller group (Box 1). In each step of the recursion and separately for each DMR, the group assignments made for individual samples are encoded in a sample assignment vector ${{\bf{v}}}$. A sample is encoded with $-{{\bf{1}}}$ or ${{\bf{1}}}$ if it belongs to the largest of the two sets ${G}_{k}$ or ${H}_{k}$; with ${{\bf{0}}}$, otherwise. If a sufficient number of samples (${N}_{\min }$) are encoded with either $-{{\bf{1}}}$ (or ${{\bf{1}}}$) or ${{\bf{0}}}$, the methylation difference $S\left({G}_{k},{H}_{k}\right)$ is added to a hash map using the assignment pattern ${{\bf{v}}}$ as a key. In this way, the methylation differences associated with a particular sample assignment pattern are summed up over all available DMRs. Subsequently, the pattern with the largest cummulative difference ${{\bf{u}}}$ is sought. If the total difference underscores the threshold ${W}_{\min }$, the samples are reported as leaves of the current branch. Otherwise, the samples are split using the pattern encoded in ${{\bf{u}}}$ and the recursion continues. During the computation, metilene^3^ stores the assignment patterns and the associated DMRs to allow the investigation of all DMRs that gave rise to the respective splits in the DMTree.

Box 1 DMTree split function $$	 {\bf{function}}\,{\rm{Split}}(E) \\ 	 \qquad {W}[{\bf{w}}]=0,\forall {\bf{w}}\in \{-1,0,1\}^{|E|} \\ 	 \qquad \,{\bf{for}}\,k\,\mathrm{in}\,{\bf{K}}\,{\bf{do}}\,\\ 	 \qquad \qquad {\bf{v}}={{\bf{0}}}^{{|E|}}\\ 	 \qquad \qquad \,{v}_{i}\leftarrow \left\{\begin{array}{cc}1,\hfill & \left|{H}_{k}\right| \, > \,\left|{G}_{k}\right|\wedge \sigma (i)\in {H}_{k} \hfill\\ -1,\hfill & \left|{H}_{k}\right|\le \left|{G}_{k}\right|\wedge \sigma (i)\in {G}_{k}\\ 0,\hfill & \,\mathrm{otherwise}\, \hfill\end{array}\,,\,i=1,\ldots,{|E|}\right. \hfill \\ 	 \qquad \qquad \,{\bf{if}}\,\left(\sum \left|\,{v}_{i}\,\right| > {N}_{\min }\right)\wedge \left({|E|}-\sum \left|\,{v}_{i}\,\right| > {N}_{\min }\right)\,{\bf{then}}\,\\ 	 \qquad \qquad \qquad {W}[{\bf{v}}] +=\left|S\left({G}_{k},{H}_{k}\right)\right|\\ 	 \qquad \qquad \,{\bf{end}}\; {\bf{if}}\,\\ 	 \qquad \,{\bf{end}}\; {\bf{for}}\,\\ 	 \qquad {\bf{u}}=\mathop{{\rm{argmax}}}\limits_{{\bf{w}}}W[{\bf{w}}]\,\\ 	 \qquad \,{\bf{if}}\,W[{\bf{u}}]\, < \,{W}_{\min }\,{\bf{then}}\,\\ 	 \qquad \qquad \,{\mathrm{report}}\,E\,{\mathrm{as}}\, {\mathrm{a}}\, {\mathrm{leaf}}\, {\mathrm{node}}\,\\ 	 \qquad \,{\bf{else}}\,\\ 	 \qquad \qquad {L}\leftarrow \left\{\sigma (i)\,|\,{{\bf{u}}}_{i}=0\right\}\\ 	 \qquad \qquad {R}\leftarrow \left\{\sigma (i)\,|\,{{\bf{u}}}_{i}\ne 0\right\}\\ 	 \qquad \qquad {\rm{Split}}(L)\\ 	 \qquad \qquad {\rm{Split}}(R)\\ 	 \qquad \,{\bf{end}}\; {\bf{if}}\,\\ 	 \,{\bf{end}}\; {\bf{function}}$$

### Simulation

In this study, we modified the methylome simulation framework from our previous work^5^ to extend its applicability to multi-condition. We simulated two CpG methylation backgrounds, each consisting of 50 samples, on human Chromosome 10 as previously described in ref. ^5^. These samples were classified into five groups: G1 (*n* = 10), G2 (*n* = 10), G3 (*n* = 10), G4 (*n* = 10), G5 (*n* = 10). Based on the five aforementioned groups, we introduced five types of DMRs, totaling 1000 DMRs, and the methylation rates $p$ within the DMRs were re-simulated under four conditions (Fig. 2a):

condition A:

9 $$p\sim {{\rm{Beta}}}\left(\beta,\alpha \right)\cdot c+{{\rm{Beta}}}\left(\alpha,\beta \right)\cdot \left(1-c\right)$$

condition B:

10 $$p\sim {{\rm{Beta}}}\left(\alpha,\beta \right)\cdot c+{{\rm{Beta}}}\left(\beta,\alpha \right)\cdot \left(1-c\right)$$

condition C:

11 $$p\sim {{\rm{Beta}}}\left(\frac{\lceil\alpha+\beta \rceil}{2},\frac{\lceil\alpha+\beta \rceil}{2}\right)$$

condition D:

12 $$p\sim {{\rm{Beta}}}\left(\beta,\alpha \right)\cdot \left[r\cdot \left(c-0.5\right)+0.5\right]+{{\rm{Beta}}}\left(\alpha,\beta \right)\cdot \left[0.5-r\cdot \left(c-0.5\right)\right]$$

here, $\alpha$ and $\beta$ are the parameters of beta distribution, $r$ is the scaling factor and $c$ is the mixing factor. We used $\alpha=40$ and $\beta=3$ (or $\alpha=3$ and $\beta=40$, randomly with a 50% probability) for background 1 and $\alpha=15$ and $\beta=5$ (or $\alpha=5$ and $\beta=15$, randomly with a 50% probability) for background 2, and randomly assign $c$ from [0.6, 0.73, 0.87, 1] to a simulated DMR.

In each DMR type, each group uses the conditions in Table 1.

**Table 1:** Conditions in DMR types I-V

	Type I	Type II	Type III	Type IV	Type V
G1	A	A	B	C	D($r=0.5$)
G2	B	A	B	C	D($r=0.5$)
G3	B	A	A	C	D($r=1$)
G4	B	B	C	A	B
G5	B	B	C	B	B

Unlike the other DMR types, for DMR type V, the re-simulation of the CpG methylation rates in G1 started from the $f{l}^{{th}}$ CpG in the DMR and stopped at the ${\left(N-{fl}\right)}^{{th}}$ CpG where $N$ is the number of CpGs in the DMR and ${fl}$ is a random number from 6 to 15.

### Purity simulation

To mimic the tumour microenvironment, we assigned each sample a simulated purity value drawn from a normal distribution with mean 0.86 and variance 0.11, derived from the TCGA-LGG + GBM cohort^49^. The final methylation rate is calculated as:

13 $${\beta }_{i}={\beta }_{{tumo}{r}_{i}}+{purit}{y}_{i}\cdot {\beta }_{{ct}{r}_{i}}$$

where ${\beta }_{{ct}{r}_{i}}$ is the background (without DMR) methylation values of sample $i$. To identify the minimal purity required for robust DMTree clustering, we further simulated datasets with purity mean of 0.8 to 0.5 and variance of 0.1, and evaluated the average Rand index of DMTree clustering results on these datasets.

### Confounding factor simulation

We introduced two confounding factors, ${C}_{1}$ and ${C}_{2}$ to increase the heterogeneity within subgroups. ${C}_{1}$ is drawn from ${unif}\left(0,1\right)$ and ${C}_{2}$ is drawn from $N\left(0.5,0.1\right)$. For each confounding factor, 500 regions were simulated to be correlated with ${C}_{1}$ or ${C}_{2}$. For each region $j$ correlated with $C$ ($C\in \{{C}_{1},{C}_{2}\}$), the methylation rate ${p}_{i,j}$ for sample $i$ is:

14 $${p}_{i,j}\sim {{\rm{Beta}}}\left(\alpha,\beta \right)\cdot {s}_{i,j}+{{\rm{Beta}}}\left(\beta,\alpha \right)\cdot \left(1-{s}_{i,j}\right)$$

where

15 $${s}_{i,j}\sim N\left(\rho \cdot \left({C}_{i}-0.5\right)+0.5,0.3\right)$$

where $\rho$ controls the correlation coefficient between the methylation of the region and the cofounding factor:

16 $$\rho \sim {unif}\left(0.1,1\right)$$

Similar to the DMR simulation, $\alpha=40$ and $\beta=3$ (or $\alpha=3$ and $\beta=40$, randomly with a 50% probability) were used for background 1 and $\alpha=15$ and $\beta=5$ (or $\alpha=5$ and $\beta=15$, randomly with a 50% probability) were used for background 2.

### Batch effect simulation

We also acknowledged that there might be a batch effect in bisulfite sequencing. In the simulation, 25 samples were assigned to batch 1, and the others were assigned to batch 2. 500 CpGs were randomly selected to be unmethylated in batch 1 and another 500 CpGs to be unmethylation in batch 2.

### Parameter of the metilene^3^

In the supervised mode, we keep minimal mean difference of a DMR $\delta$ to 0.1 as the original metilene^5^. In the unsupervised mode, we tested different combinations of the parameters: minimal mean difference of a DMR $\delta \in \{0.1,0.25,0.5\}$, minimal number of samples in a cluster ${N}_{\min }$ from 2 to 6, and minimal cumulative difference ${W}_{\min }\in \{1,2,5,10,20\}$. To identify an optimal parameter set, we performed grid-search of these parameters on background 1 and background 2, and kept parameter sets that use the same minimal number of samples and led to consistent clustering results between two backgrounds. Five parameter sets were kept for the background 1 and one parameter set was kept for the background 2. The parameter sets were then ranked by $\delta$ and ${W}_{\min }$ in descending order and the first one was set as the default parameter of metilene^3^. For background 1, $\delta=0.5,{N}_{\min }=6,{W}_{\min }=10$ were used, and $\delta=0.25,{N}_{\min }=6,{W}_{\min }=1$ were used for background 2.

Considering that real-world datasets vary in number of samples and number of available CpGs ${n}_{{CpG}}$ (normalized to the number of simulated CpGs ${n}_{{simulatedCpG}}$), we proposed a formula to estimate the optimal ${W}_{\min }$ with:

17 $${W}^{{\prime} }=\alpha \cdot \frac{{n}_{{CpG}}}{{n}_{{simulatedCpG}}}$$

where $\alpha$ is a coefficient to take the number of samples in the real-world dataset $n$ into account by comparing to the number of samples simulated ${n}_{{sim}}$ (${n}_{{sim}}=50$):

18 $$\alpha=1+\frac{{n}_{{sim}}-1}{n-1}\cdot \left({W}_{{default}}-1\right)$$

Here ${W}_{{default}}$ would be 10 for the background 1 and 1 for the background 2. In practice, metilene^3^ will first run with the first parameter set (optimal for background 1) and if no cluster can be found, the second parameter set (optimal for background 2) will be used. In addition, to simplify the parameter, ${W}_{\min }$ will be set to 1 if ${W}^{{\prime} } < 5$ and will be set to 100 if ${W}^{{\prime} }\ge 50$, otherwise, ${W}_{\min }$ will be set to 10.

### Benchmarking on DMR identification

We tested metilene^3^, wgbstools (version 0.2.2)^13^, SMART (version 2.2.8)^12^ and methylscore^15^ using simulated datasets on a Linux computing cluster. All DMR callers were executed with default parameters, except for the number of threads. Sensitivity, precision and Jaccard index were calculated as follows:

19 $${sensitivity}=\frac{\sum {{Length}}_{{overlapped}}}{\sum {{Length}}_{{simulated}}}$$

20 $${precision}=\frac{\sum {{Length}}_{{overlapped}}}{\sum {{Length}}_{{predicted}}}$$

21 $${Jaccard}=\frac{\sum {{Length}}_{{overlapped}}}{\sum {{Length}}_{{predicted}}+\sum {{Length}}_{{simulated}}-\sum {{Length}}_{{overlapped}}}$$

The overlaps between predicted DMRs and simulated DMRs were computed using pybedtools^50^.

For local clustering, accuracy is defined as the proportion of DMRs for which the predicted group (from the predicted DMR with the largest overlap with the simulated DMR) matches the simulated group.

For time and memory usage benchmarking, DMR calling was performed using both a single core and ten cores. Since SMART does not offer a parameter to control the number of threads, it was only tested with ten cores. The workflow of methylscore could not be executed in a single run, and thus the time and memory usage could not be benchmarked.

The unsupervised mode of metilene^3^ was tested on the simulated datasets using default parameters.

### Benchmarking on clustering

We compared DMTree to unsupervised clustering methods commonly used in DNA methylation data analysis, including hierarchical clustering and K-means clustering^51^. We first applied DMTree, hierarchical clustering, and K-means clustering based on the first two principal components to the simulated dataset. To further measure the robustness, and the sensitivity of small group detection, we stochastically sampled $n$ ($n$ from one to ten) samples from group G1 and $40-n$ samples from other groups, and repeated for 50 times. For hierarchical clustering and K-means clustering, we evaluated the results under three to seven clusters. For metilene^3^, as the number of clusters is automatically determined, we evaluated the results under different minimal number of samples in a cluster, ${N}_{\min }$, from two to six. The performance of the clustering methods was measured by the average Rand index using scikit-learn^52^, compared to the simulated labels. The sensitivity of small group detection was measured by the average Rand index among samples from G1 and G2, as G2 is the most similar group to G1.

### Biological datasets

The normal human cell types WGBS data were obtained from Loyfer et al.^13^ (GSE186458 [https://www.ncbi.nlm.nih.gov/geo/query/acc.cgi?acc=GSE186458]), with only CpGs having a minimum of 10 reads retained. The glioma WGBS and RNA data^24^ were downloaded from GEO database (GSE121723 [https://www.ncbi.nlm.nih.gov/geo/query/acc.cgi?acc=GSE121723]). The cell-free DNA in cerebrospinal fluid data^26^ were downloaded from GEO database (GSE142241 [https://www.ncbi.nlm.nih.gov/geo/query/acc.cgi?acc=GSE142241]). The pancreatic ductal adenocarcinoma (PDAC) WGBS data were extracted from the RData file from Lo et al.^25^ (GSE222147 [https://www.ncbi.nlm.nih.gov/geo/query/acc.cgi?acc=GSE222147]) and were converted to a tab-separated file with 3 decimal places using pandas^53^. Pancreatic RNA data^42^ were obtained from GEO database (GSE210351 [https://www.ncbi.nlm.nih.gov/geo/query/acc.cgi?acc=GSE210351]) and UCSC Xena^43^ [https://xena.ucsc.edu/]. All bigWig files were converted to a tab-separated file with 3 decimal places using UCSC bigWigToBedGraph^54^ and bedtools^55^.

For the normal human cell types WGBS dataset, ${N}_{\min }$ was set to 3. For the PDAC dataset, the glioma dataset, and the cerebrospinal fluid cell-free DNA dataset, ${N}_{\min }$ was set to 4. As the cerebrospinal fluid cell-free DNA dataset contains only nine samples, the *p*-value filter for DMRs was not applied and we included all DMRs in the Supplementary Data. Parameters not explicitly mentioned were set to their default values.

### Visualization

For the DMTree visualization, the DMTree was first saved in Newick tree format, and the module Phylo from Biopython^56^ and matplotlib^57^ were used to visualize trees. The heatmaps showing DMRs associated with DMTree splits were visualized with seaborn^58^. Package scikit-learn^52^ was used to perform principal component analysis (PCA), and the first two components were visualized with seaborn. DNA methylation at motifs were visualized with EnrichedHeatmap^59^.

### DMR annotation

DMRs were annotated with the function’annotatePeak’ from ChIPseeker^60^. In this study, the parameter’TxDb’ was set to’TxDb.Hsapiens.UCSC.hg19.knownGene’, the parameter’tssRegion’ was set to’c(-3000, 1000)’, and the parameter’annoDb’ was set to’org.Hs.eg.db’.

### Consensus score

We first define a set of DMRs $K\left(X,Y,\theta \right)$, where $X$ can be either neoplasia or PDAC, $Y$ can be either acini or duct, and $\theta$ is an absolute methylation difference threshold. $K\left(X,Y,\theta \right)$ is a subset of DMRs with:

### Acini and ducts are locally clustered into hypo- or hyper-methylated and cannot be in the same cluster

- $X$ is locally clustered into hypo- or hyper-methylated.
- the absolute methylation difference is greater than $\theta$.

The consensus score is calculated as:

22 $${score}\left(X,Y\right)=\frac{\left|K\left(X=Y,\theta \right)\right|}{\left|K\left(X,Y,\theta \right)\right|}$$

Here $K\left(X=Y,\theta \right)$ is a subset of $K\left(X,Y,\theta \right)$ where $X$ and $Y$ are in the same local cluster.

### Motif analysis

Significant DMRs were defined as supervised DMRs with an absolute mean difference greater than 0.5. Among these, those exhibiting a consistent pattern with a DMTree split were classified as the positive set, while those displaying a reversed pattern were designated as the negative set. Motif enrichment analysis was performed using HOMER^61^ on two target sets: the positive set and the negative set. The region size was set to 250 bp, and the reference genome was hg19. Background was set to all significant DMRs excluding the target sets.

### Differentially expressed genes analysis

For the glioma cohort, we first converted the RNAseq coverage signals (bigwig files) to gene-level values using multiBigwigSummary^62^ and gencode.v19.annotation^63^. Next, we selected genes with at least 50% data points with at least one read on average, and used DESeq2^64^ to compare gene expression between cluster B versus cluster C. GSEA was performed on the fold change of genes from DESeq2, with the function’prerank’ from package gseapy and hallmark gene sets^65^.

### Gene Set Enrichment Analysis

Gene Set Enrichment Analysis (GSEA) was performed with the Python package’gseapy’^66^.

### Reporting summary

Further information on research design is available in the Nature Portfolio Reporting Summary linked to this article.

## Supplementary information

Supplementary Information

Description of Additional Supplementary Files

Supplementary Datasets 1-9

Supplementary Software 1

Supplementary Software 2

Reporting Summary

Transparent Peer Review file

## Source data

Source Data

## Data Availability

The simulated test data are deposited on Zenodo^67^ [10.5281/zenodo.20021217]. Previously published, public WGBS data were used for testing. This includes the tissue atlas and blood data (GSE186458 [https://www.ncbi.nlm.nih.gov/geo/query/acc.cgi?acc=GSE186458]), pancreas data (GSE222147 [https://www.ncbi.nlm.nih.gov/geo/query/acc.cgi?acc=GSE222147]), glioma data (GSE121723 [https://www.ncbi.nlm.nih.gov/geo/query/acc.cgi?acc=GSE121723]), as well as the medulloblastoma tumor and cfDNA data (GSE142241 [https://www.ncbi.nlm.nih.gov/geo/query/acc.cgi?acc=GSE142241]). Public RNA data were used for validation and include pancreatic tissues (GSE210351 [https://www.ncbi.nlm.nih.gov/geo/query/acc.cgi?acc=GSE210351]) and TCGA/GTEx pancreatic cancer and matched normal samples from UCSC Xena [https://xena.ucsc.edu/]. Source data are provided with this paper.

## Code availability

Metilene^3^ code with test data, as well as documentation, is available under the following link: https://github.com/zzhu1372/metilene3 and https://github.com/zzhu1372/metilene3-fig, and in the Supplementary Software 1 and 2, under license GNU General Public License v2.0. The specific version of the code associated with this publication is archived in Zenodo^68^ and is accessible via [10.5281/zenodo.20025001].
