# Supplementary material for: metilene3: identifying DMRs across multiple conditions with auto-classification
Source: Nat Commun. 2026 Jul 4;17:5848. doi: 10.1038/s41467-026-74931-y (PMC13333024; doi:10.1038/s41467-026-74931-y)
Supplement: Supplementary file 1 — Supplementary Information [file 41467_2026_74931_MOESM1_ESM.pdf]

Supplementary Figures

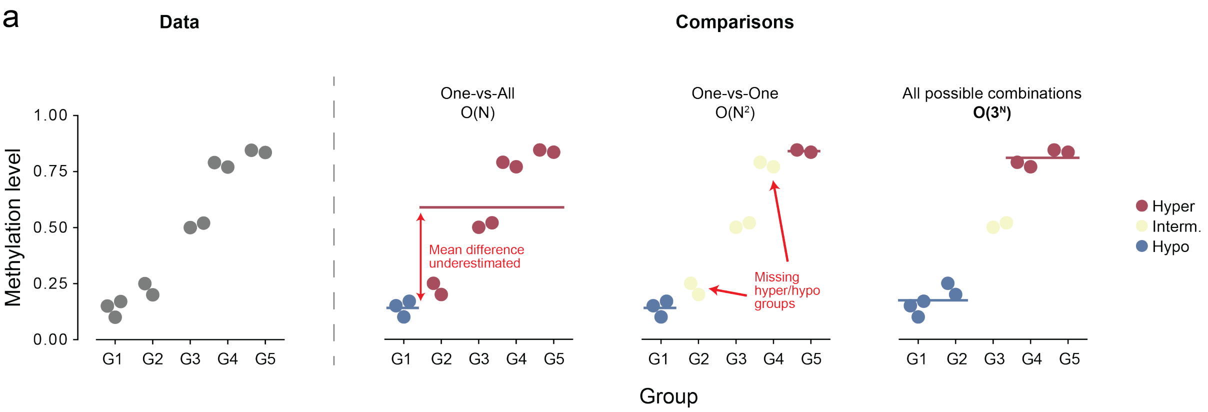

Supplementary Fig. 1: Complexity of DMR calling in multi-group comparisons.

a) Schematic of the complexity of calling DMRs between more than two groups. (Left) Example for average regional methylation rates across five groups, with each dot representing a replicate. (Right) Three approaches for converting a multi-group DMR comparison into pairwise comparisons: 1) One group vs. all other groups - may underestimate absolute mean differences by averaging out group-specific signals. 2) One group vs. one group - excludes information from additional groups, potentially missing relevant groups by excluding them from the hyper/hypo sets. 3) Group set vs. group set - tests all possible combinations of group sets, offering the most comprehensive comparison. However, the number of possible mean difference calculations increases exponentially, following $\frac{3^N-(2\times2^N-1)}{2}$, e.g., resulting in 90 comparisons for N=5 groups and 28,501 for N=10 groups. In unsupervised mode, each sample is treated as an individual group, further increasing the complexity of DMR identification. Blue/red/yellow dots indicate groups defined as hypo/hyper/intermediate in the respective comparison.

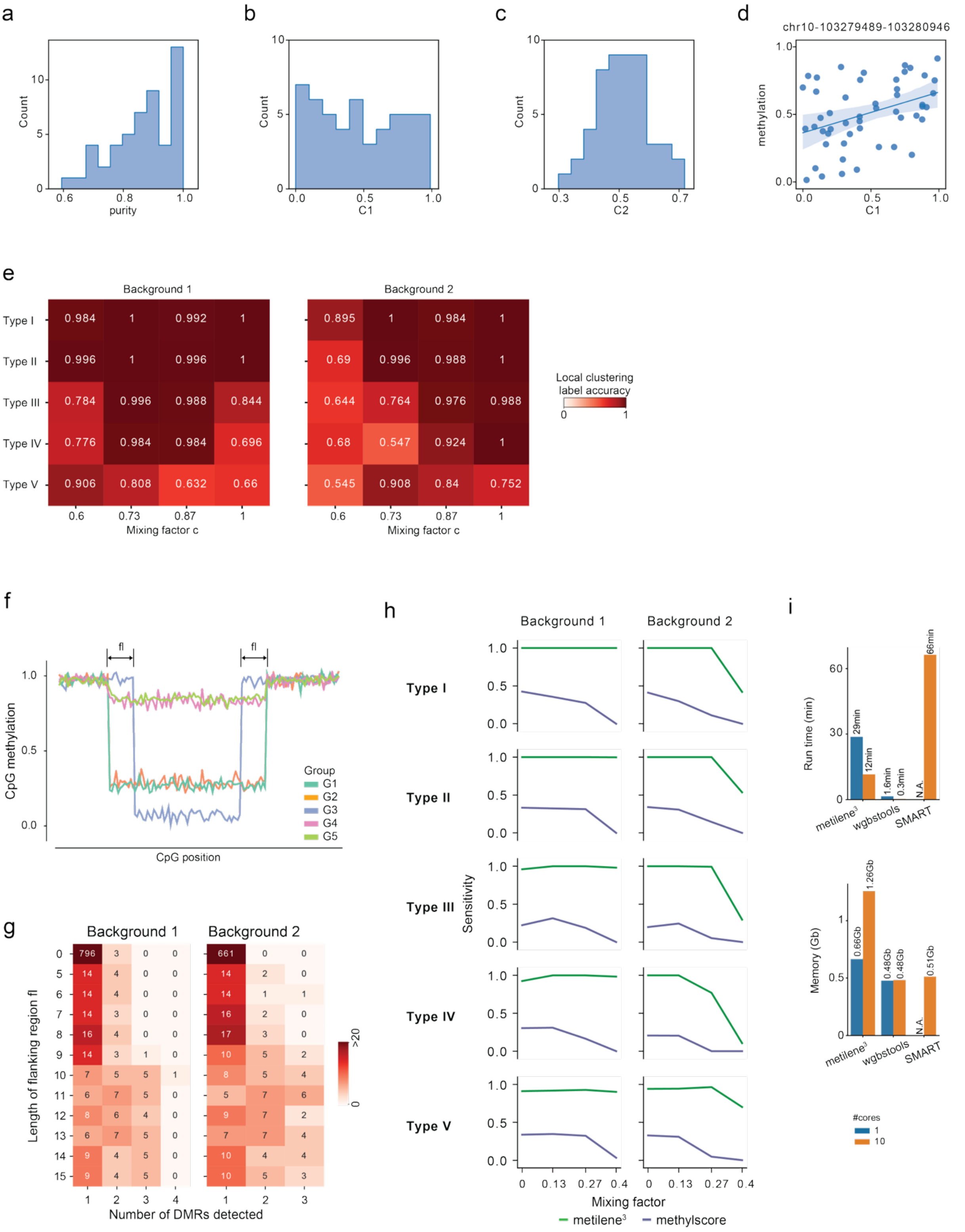

Supplementary Fig. 2: Methylome simulation and evaluation of metilene[3].

a) Distribution of simulated sample purity.
b) Distribution of confounding factor C1, which is drawn from a uniform distribution.
c) Distribution of confounding factor C2, which is drawn from a normal distribution.
d) Example of a confounding factor-correlated region. In this example, the methylation ratio of the region is positively correlated to the confounding factor C1.

e) Accuracy of local clustering across DMR types and mixing factors. Local clustering accuracy is shown for all five DMR types across two background complexities (1 and 2) and varying mixing factors (c). While simpler DMR structures (Types 1 and 2) enable accurate group assignments largely independent of the mixing factor, more complex DMRs lead to reduced clustering accuracy in local clustering. Smaller mixing factors correspond to a smaller mean difference between the hypomethylated and hypermethylated groups.

f) Schematic representation of complex DMR type V, characterized by shuffled DMRs. In this scenario, DMR borders shift dynamically between different one-on-one group comparisons. The flanking region (fl) represents these shifts, illustrating how DMR boundaries vary across samples.

g) Detection of DMRs in regions with flanking shifts in methylation changes. The number of detected DMRs depends on the length of the flanking regions: If only the central DMR is detected, then n = 1 DMR. If the central DMR is detected along with one flanking region, while the second flanking region is identified as a separate DMR, then n = 2 DMRs. If all three regions are identified separately, n = 3 DMRs. This analysis highlights how metilene[3] adapts to varying DMR complexities and shifts in flanking region lengths.

h) Sensitivity of metilene[3] and methylscore in unsupervised DMR detection under varying conditions.

i) Computational performance is assessed on a single core and across 10 cores, highlighting efficiency in runtime and memory usage.
Source data are provided as a Source Data file.

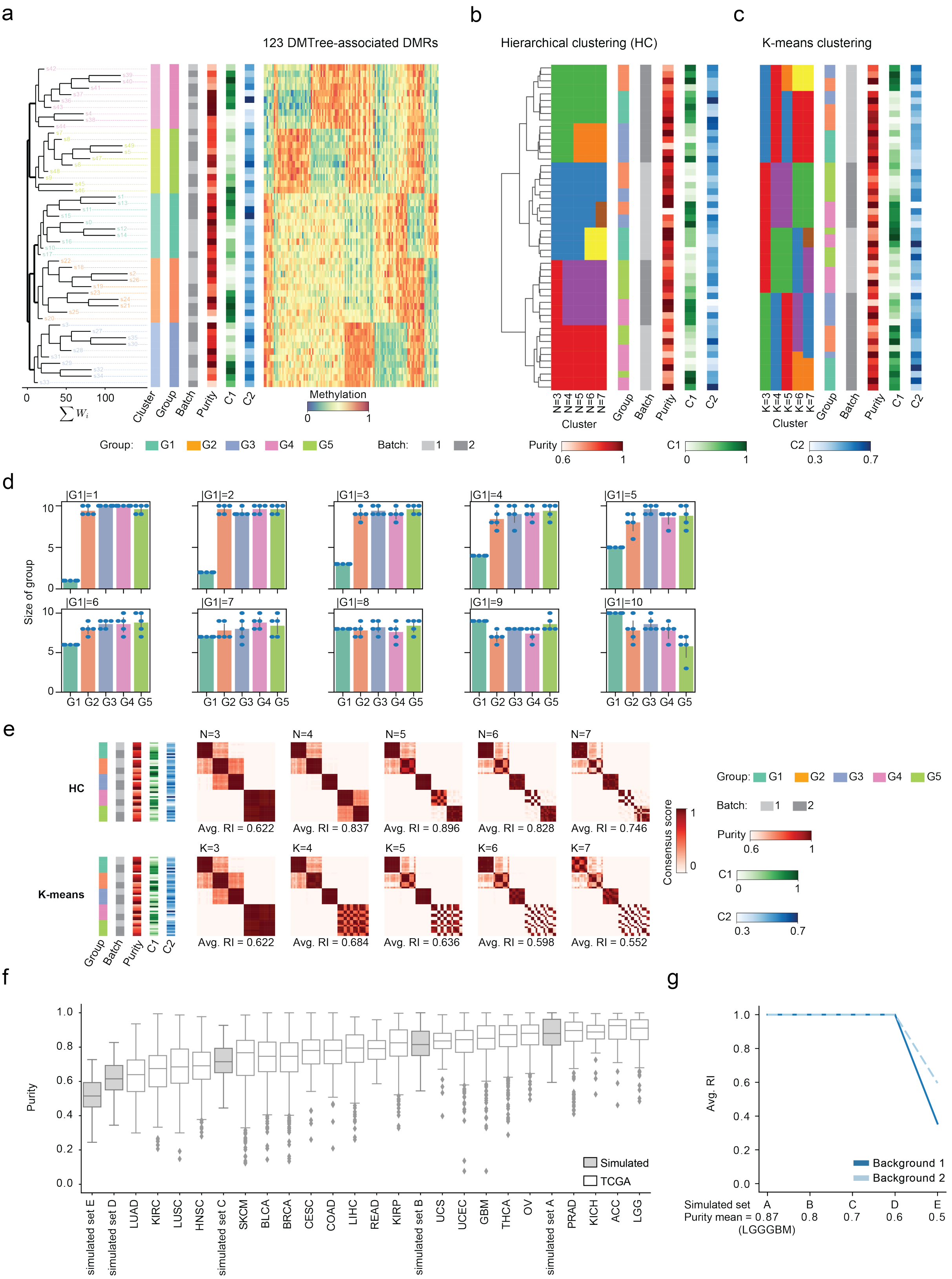

Supplementary Fig. 3: Benchmarking DMTree.

a) DMTree clustering (left) and corresponding heatmap (right) based on DMTree-associated unsupervised DMRs in the noisy background 2. The inferred group assignments from DMTree clustering align with the simulated group assignments across all samples. Annotation bars (middle): DMTree-based clustering, with group

memberships indicated by light-colored bars, and simulated sample annotations including group, batch, purity, and confounding factors C1 and C2. The final cluster splits in the DMTree are highlighted in bold.

b) Hierarchical clustering (HC) of samples based on the top 1% variable CpGs, with the number of clusters from three to seven, in the noisy background 2.

c) K-means clustering of samples based on the first two principal components of the top 1% variable CpGs, with K ranging from three to seven, in the noisy background 2.

d) Sizes of five simulated groups in the repeated sampling experiment. The size of group G1, |G1|, is first determined (from one to ten), and the rest of the samples (40-|G1|) are randomly selected from the other groups. The error bar shows 95% confidence intervals.

e) Consensus matrix across 50 resampled runs. Samples are ordered by simulated groups, batches, and purity. DMTree is evaluated for different minimum cluster sample sizes (n). HC and K-means using DMTree used DMRs are evaluated for different numbers of clusters (N for HC and K for K-means). Avg. RI: average Rand index.

f) Purity ranges of different groups in the TCGA cohort (number of samples: LUAD=530, KIRC=539, LUSC=504, HNSC=528, SKCM=470, BLCA=411, BRCA=1096, CESC=305, COAD=469, LIHC=375, READ=167, KIRP=291, UCS=57, UCEC=551, GBM=605, THCA=503, OV=579, PRAD=498, KICH=66, ACC=80, LGG=520) and of five simulated groups (n=50) ranging across the entire TCGA purity spectrum, with group E showing on average lower purity levels (0.5) than all TCGA groups (0.6 and above). Boxes show the first and third quartiles with the median at the centre and whiskers extend to the minimum and maximum within 1.5x the inter-quartile range.

g) Average Rand index of simulated sets A to E using DMTree, under varying purity levels (0.87, 0.8, 0.7, 0.6, 0.5). Avg. RI: average Rand index.
Source data are provided as a Source Data file.

a

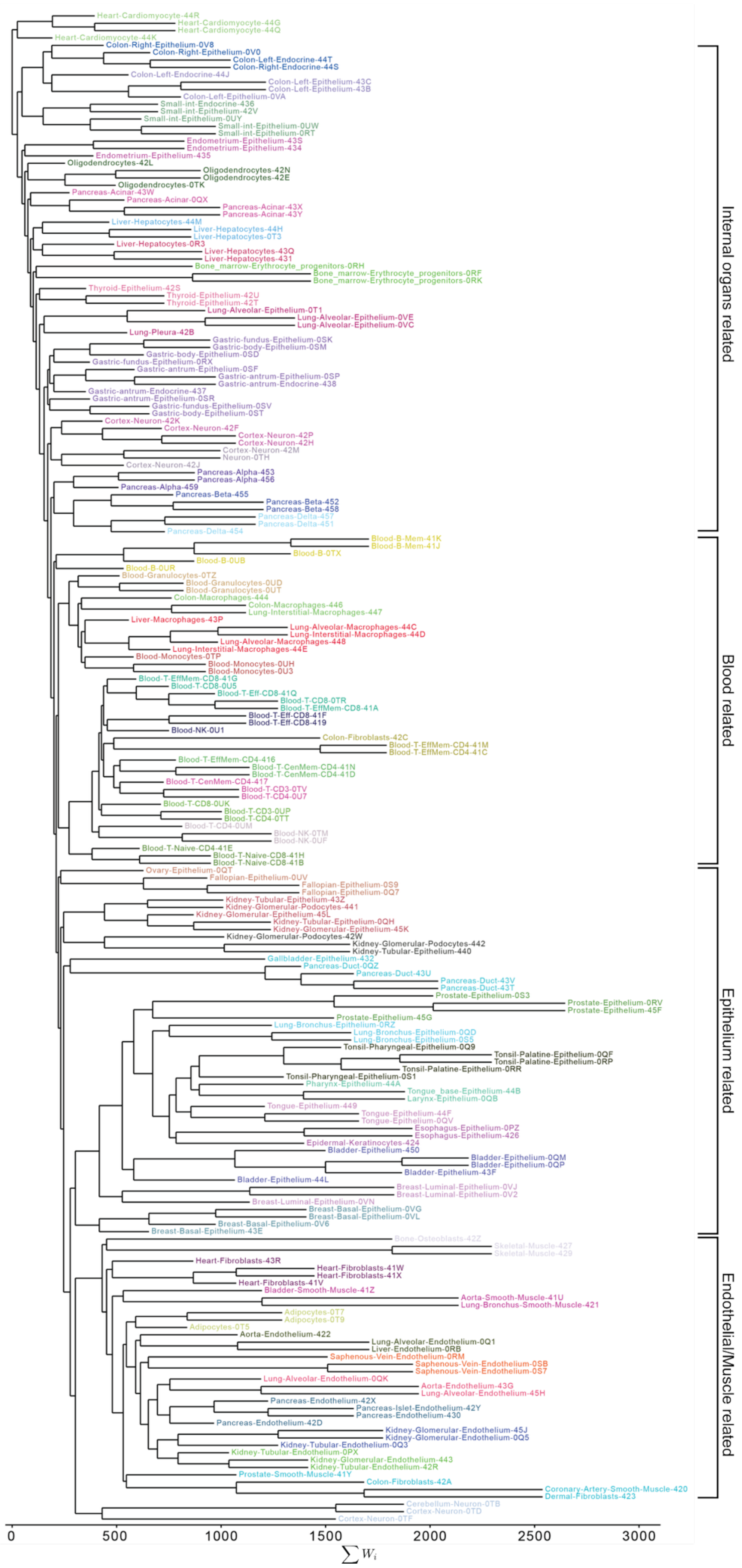

Heart-Cardiomyocyte-44R
Heart-Cardiomyocyte-44G
Heart-Cardiomyocyte-44Q
Heart-Cardiomyocyte-44K
Colon-Right-Epithelium-0V8
Colon-Right-Epithelium-0V0
Colon-Left-Endocrine-44T
Colon-Right-Endocrine-44S
Colon-Left-Endocrine-44J
Colon-Left-Epithelium-43C
Colon-Left-Epithelium-43B
Colon-Left-Epithelium-0VA
Small-int-Endocrine-436
Small-int-Epithelium-42V
Small-int-Epithelium-0UY
Small-int-Epithelium-0UW
Small-int-Epithelium-0RT
Endometrium-Epithelium-43S
Endometrium-Epithelium-434
Endometrium-Epithelium-435
Oligodendrocytes-42L
Oligodendrocytes-42N
Oligodendrocytes-42E
Oligodendrocytes-0TK
Pancreas-Acinar-43W
Pancreas-Acinar-0QX
Pancreas-Acinar-43X
Pancreas-Acinar-43Y
Liver-Hepatocytes-44M
Liver-Hepatocytes-44H
Liver-Hepatocytes-0T3
Liver-Hepatocytes-0R3
Liver-Hepatocytes-43Q
Liver-Hepatocytes-431
Bone_marrow-Erythrocyte_progenitors-0RH
Bone_marrow-Erythrocyte_progenitors-0RF
Bone_marrow-Erythrocyte_progenitors-0RK
Thyroid-Epithelium-42S
Thyroid-Epithelium-42U
Thyroid-Epithelium-42T
Lung-Alveolar-Epithelium-0T1
Lung-Alveolar-Epithelium-0VE
Lung-Alveolar-Epithelium-0VC
Lung-Pleura-42B
Gastric-fundus-Epithelium-0SK
Gastric-body-Epithelium-0SM
Gastric-body-Epithelium-0SD
Gastric-fundus-Epithelium-0RX
Gastric-antrum-Epithelium-0SF
Gastric-antrum-Epithelium-0SP
Gastric-antrum-Endocrine-438
Gastric-antrum-Endocrine-437
Gastric-antrum-Epithelium-0SR
Gastric-fundus-Epithelium-0SV
Gastric-body-Epithelium-0ST
Cortex-Neuron-42K
Cortex-Neuron-42F
Cortex-Neuron-42P
Cortex-Neuron-42H
Cortex-Neuron-42M
Neuron-0TH
Cortex-Neuron-42J
Pancreas-Alpha-453
Pancreas-Alpha-456
Pancreas-Alpha-459
Pancreas-Beta-455
Pancreas-Beta-452
Pancreas-Beta-458
Pancreas-Delta-457
Pancreas-Delta-451
Pancreas-Delta-454
Blood-B-Mem-41K
Blood-B-Mem-41J
Blood-B-0TX
Blood-B-0UB
Blood-B-0UR
Blood-Granulocytes-0TZ
Blood-Granulocytes-0UD
Blood-Granulocytes-0UT
Colon-Macrophages-444
Colon-Macrophages-446
Lung-Interstitial-Macrophages-447
Liver-Macrophages-43P
Lung-Alveolar-Macrophages-44C
Lung-Interstitial-Macrophages-44D
Lung-Alveolar-Macrophages-448
Lung-Interstitial-Macrophages-44E
Blood-Monocytes-0TP
Blood-Monocytes-0UH
Blood-Monocytes-0U3
Blood-T-EffMem-CD8-41G
Blood-T-CD8-0U5
Blood-T-Eff-CD8-41Q
Blood-T-CD8-0TR
Blood-T-EffMem-CD8-41A
Blood-T-Eff-CD8-41F
Blood-T-Eff-CD8-419
Blood-NK-0U1
Colon-Fibroblasts-42C
Blood-T-EffMem-CD4-41M
Blood-T-EffMem-CD4-41C
Blood-T-EffMem-CD4-416
Blood-T-CenMem-CD4-41N
Blood-T-CenMem-CD4-41D
Blood-T-CenMem-CD4-417
Blood-T-CD3-0TV
Blood-T-CD4-0U7
Blood-T-CD8-0UK
Blood-T-CD3-0UP
Blood-T-CD4-0TT
Blood-T-CD4-0UM
Blood-NK-0TM
Blood-NK-0UF
Blood-T-Naive-CD4-41E
Blood-T-Naive-CD8-41H
Blood-T-Naive-CD8-41B
Ovary-Epithelium-0QT
Fallopian-Epithelium-0UV
Fallopian-Epithelium-0S9
Fallopian-Epithelium-0Q7
Kidney-Tubular-Epithelium-43Z
Kidney-Glomerular-Podocytes-441
Kidney-Glomerular-Epithelium-45L
Kidney-Tubular-Epithelium-0QH
Kidney-Glomerular-Epithelium-45K
Kidney-Glomerular-Podocytes-42W
Kidney-Glomerular-Podocytes-442
Kidney-Tubular-Epithelium-440
Gallbladder-Epithelium-432
Pancreas-Duct-0QZ
Pancreas-Duct-43U
Pancreas-Duct-43V
Pancreas-Duct-43T
Prostate-Epithelium-0S3
Prostate-Epithelium-0RV
Prostate-Epithelium-45F
Prostate-Epithelium-45G
Lung-Bronchus-Epithelium-0RZ
Lung-Bronchus-Epithelium-0QD
Lung-Bronchus-Epithelium-0S5
Tonsil-Pharyngeal-Epithelium-0Q9
Tonsil-Palatine-Epithelium-0QF
Tonsil-Palatine-Epithelium-0RP
Tonsil-Palatine-Epithelium-0RR
Tonsil-Pharyngeal-Epithelium-0S1
Pharynx-Epithelium-44A
Tongue_base-Epithelium-44B
Larynx-Epithelium-0QB
Tongue-Epithelium-449
Tongue-Epithelium-44F
Tongue-Epithelium-0QV
Esophagus-Epithelium-0PZ
Esophagus-Epithelium-426
Epidermal-Keratinocytes-424
Bladder-Epithelium-450
Bladder-Epithelium-0QM
Bladder-Epithelium-0QP
Bladder-Epithelium-43F
Bladder-Epithelium-44L
Breast-Luminal-Epithelium-0VJ
Breast-Luminal-Epithelium-0V2
Breast-Luminal-Epithelium-0VN
Breast-Basal-Epithelium-0VG
Breast-Basal-Epithelium-0VL
Breast-Basal-Epithelium-0V6
Breast-Basal-Epithelium-43E
Bone-Osteoblasts-42Z
Skeletal-Muscle-427
Skeletal-Muscle-429
Heart-Fibroblasts-43R
Heart-Fibroblasts-41W
Heart-Fibroblasts-41X
Heart-Fibroblasts-41V
Bladder-Smooth-Muscle-41Z
Aorta-Smooth-Muscle-41U
Lung-Bronchus-Smooth-Muscle-421
Adipocytes-0T7
Adipocytes-0T9
Adipocytes-0T5
Aorta-Endothelium-422
Lung-Alveolar-Endothelium-0Q1
Liver-Endothelium-0RB
Saphenous-Vein-Endothelium-0RM
Saphenous-Vein-Endothelium-0SB
Saphenous-Vein-Endothelium-0S7
Lung-Alveolar-Endothelium-0QK
Aorta-Endothelium-43G
Lung-Alveolar-Endothelium-45H
Pancreas-Endothelium-42X
Pancreas-Islet-Endothelium-42Y
Pancreas-Endothelium-430
Pancreas-Endothelium-42D
Kidney-Glomerular-Endothelium-45J
Kidney-Glomerular-Endothelium-0Q5
Kidney-Tubular-Endothelium-0Q3
Kidney-Tubular-Endothelium-0PX
Kidney-Glomerular-Endothelium-443
Kidney-Tubular-Endothelium-42R
Prostate-Smooth-Muscle-41Y
Colon-Fibroblasts-42A
Coronary-Artery-Smooth-Muscle-420
Dermal-Fibroblasts-423
Cerebellum-Neuron-0TB
Cortex-Neuron-0TD
Cortex-Neuron-0TF
0
500
1000
1500
2000
2500
3000
$\sum W_i$
Internal organs related
Blood related
Epithelium related
Endothelial/Muscle related

Supplementary Fig. 4: DMTree clustering of primary human cell types based on unsupervised DMR calling.

a) DMTree clustering of 205 WGBS samples representing 77 primary cell types across 39 broader cell type categories. Unsupervised DMR calling assigned the 205 samples to 56 clusters, which largely reflect the developmental lineage relationships of the respective cell types (parameters: minimum samples per cluster: 3, minimum absolute DMR difference: 0.5, minimum total methylation difference W: 10).
Source data are provided as a Source Data file.

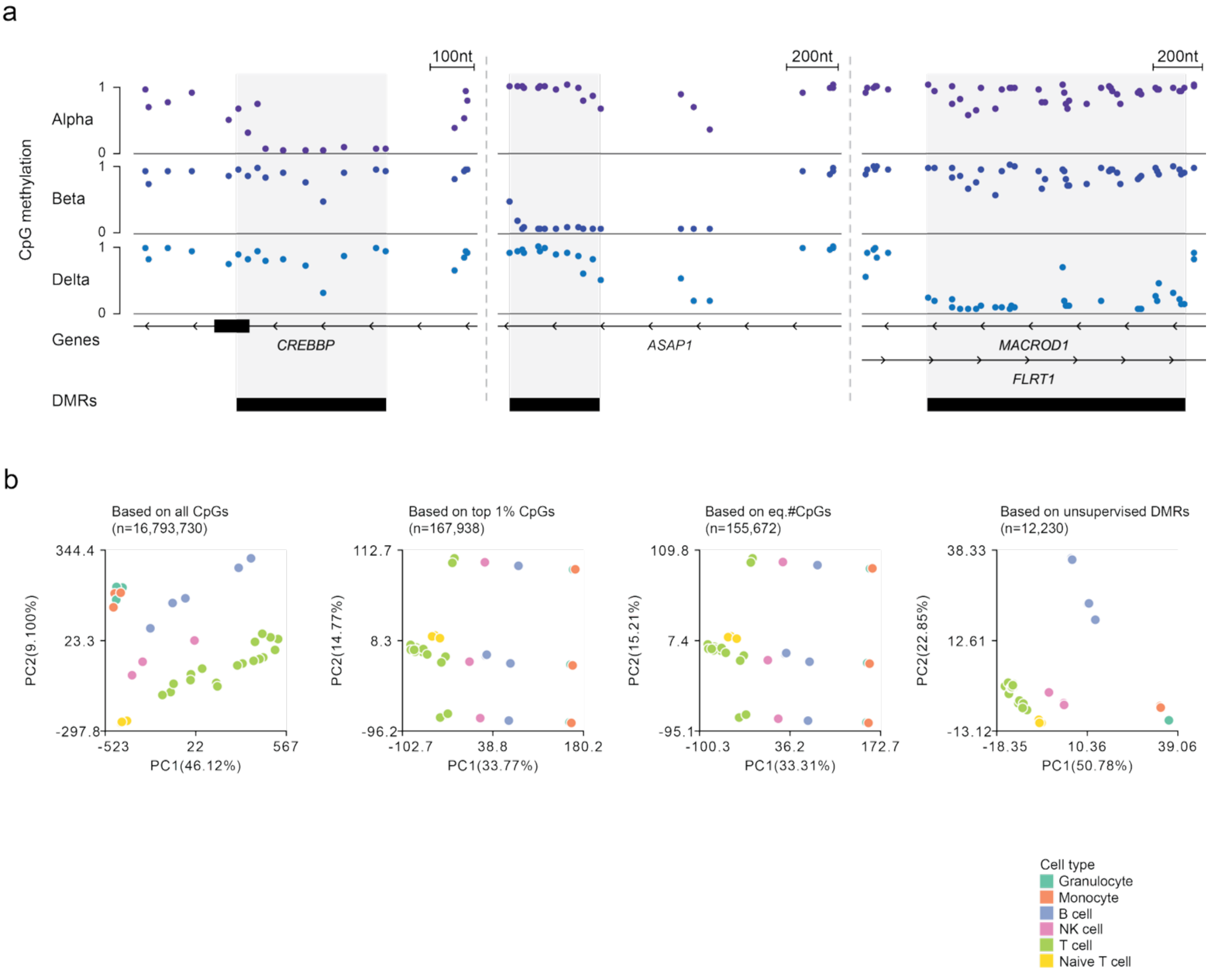

Supplementary Fig. 5: Unsupervised DMR calling detects cell-type-specific methylation patterns.

a) CpG methylation patterns at three DMRs covering CREBBP, ASAP1, and FLRT1 in pancreatic islet cells. CREBBP is specifically hypomethylated in alpha cells, ASAP1 shows beta cell-specific hypomethylation, and FLRT1 exhibits a delta cell-specific hypomethylation pattern. These DMRs support the clear separation of alpha, beta, and delta cells in the DMTree clustering of all 205 samples.

b) Principal Component Analysis (PCA) of CpG methylation in blood samples. (Left) PCA based on all CpGs shows a wide distribution of B and T cells along PC1 and a mixture of Granulocytes and Monocytes, indicating incomplete separation of myeloid and lymphoid lineages. (Middle left and middle right) PCA based on the most variable CpGs (middle left: top 1%, middle right: equivalent number of CpGs used to build DMTree) stratifies samples by a higher level of cell type, but also fails to group samples from the same detailed cell type. In addition, samples are stratified by the second PC, which shows no correlation with cell type. (Right) PCA based on unsupervised DMRs results in a clear separation of all six blood cell types, with PC1 distinguishing lymphoid from myeloid lineages and PC2 separating B cells from other lymphocytic subtypes.

Source data are provided as a Source Data file.

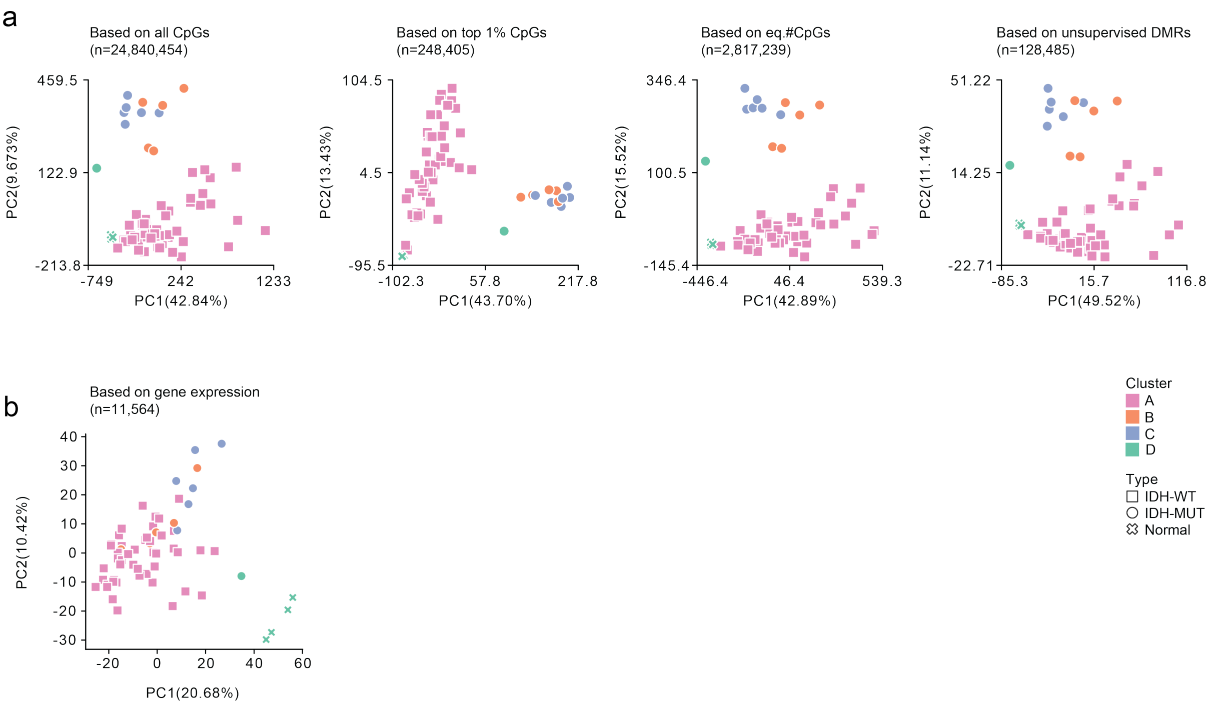

c

Rank	Motif	P-value	% of Targets	% of Background	STD(Bg STD)	Best Match
1	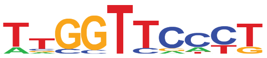	10^{-27}	3.66%	0.15%	72.3bp (8.5bp)	ZNF766/MA2098.1/Jaspar(0.633)
2	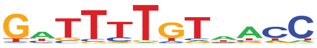	10^{-27}	3.66%	0.15%	69.3bp (101.3bp)	CDX1/MA0878.3/Jaspar(0.678)
3	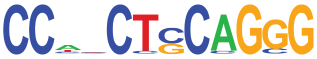	10^{-22}	3.84%	0.22%	71.2bp (34.1bp)	BORIS(Zf)/K562-CTCFL-ChIP-Seq(GSE32465)/Homer(0.637)

Supplementary Fig. 6: Detection of outlier samples using unsupervised DMR calling.

a) PCA based on all CpGs, most variable CpGs (middle left: top 1%, middle right: equivalent number of CpGs used to build DMTree), and PCA based on unsupervised DMRs all separate IDH-MUT from IDH-WT and control samples. All models appear to capture normal versus glioblastoma-specific methylation patterns, as well as methylation heterogeneity within glioblastomas. In both analyses, the IDH-MUT sample AK015 behaves as an outlier, which is a pattern that would have been missed using predefined sample groups based on diagnosis.

b) PCA based on gene expression of the top 20% variable genes shows AK015 clusters together with cancer samples instead of normal controls, but is also closer to normal tissues compared to other tumors.

c) Top three enriched *de novo* motifs in DMRs associated with the split distinguishing cluster B from cluster C.
Source data are provided as a Source Data file.

a

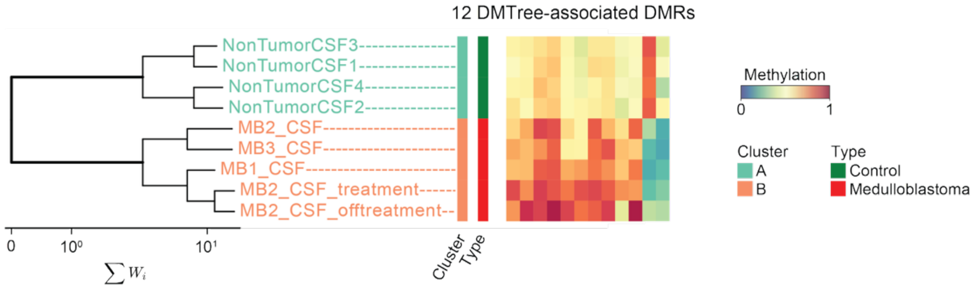

Supplementary Fig. 7: DMTree of the medulloblastoma dataset.

a) DMTree clustering (left) and corresponding heatmap (right) based on DMTree-associated unsupervised DMRs in 9 cerebrospinal fluid (CSF) samples of circulating cell-free DNA (cfDNA) from Medulloblastoma and non-tumor patients. A single split is detected, clearly separating Medulloblastoma-derived cfDNA samples from non-tumor controls, regardless of treatment status (parameters: minimum samples per cluster: 4, minimum absolute DMR difference: 0.25, minimum total methylation difference W: 1) Source data are provided as a Source Data file.

a

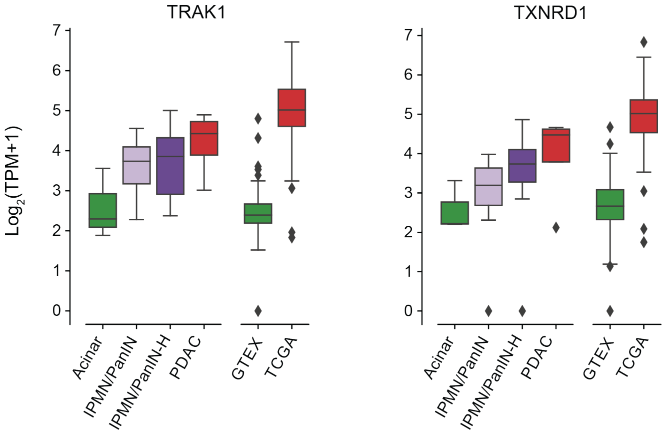

Supplementary Fig. 8: Expression of DMR-associated genes in pancreatic samples.

a) Expression of TXNRD1 and TRAK1 across acinar (n=3), IPMN/PanIN (n=18), high-grade IPMN/PanIN (IPMN/PanIN-H, n=16), PDAC (n=4), GTEx-pancreas (n=167), and TCGA's pancreas adenocarcinoma (PAAD, n=179), from GSE210351[1] and UCSC Xena[2]. Both genes show significantly higher expression levels in cancer compared to their benign counterparts. Boxes show the first and third quartiles with the median at the centre and whiskers extend to the minimum and maximum within 1.5x the inter-quartile range.
Source data are provided as a Source Data file.

a

PDAC-hyper DMRs

Rank	Motif	Name	P-value	# Target	% of Targets	# Background	% of Background
1		ZNF317	10^{-2}	5.0	4.00%	36.0	0.72%
2		Six1	10^{-2}	7.0	5.60%	80.5	1.61%
3		NRSF	10^{-2}	3.0	2.40%	13.7	0.27%

PDAC-hypo DMRs

Rank	Motif	Name	P-value	# Target	% of Targets	# Background	% of Background
1		RORg	10^{-21}	44.0	8.40%	101.9	1.25%
2		Smad2	10^{-17}	52.0	9.92%	187.2	2.31%
3		NFKB2	10^{-16}	117.0	22.33%	809.8	9.97%
4		ERb	10^{-14}	63.0	12.02%	314.0	3.87%
5		NFKB	10^{-11}	83.0	15.84%	555.2	6.84%
6		NFATC1	10^{-11}	106.0	20.23%	815.5	10.04%

b

DMR type		NFKB2-motif Yes	NFKB2-motif No	NFATC1-motif Yes	NFATC1-motif No	NFKB2+NFATC1-motif Yes	NFKB2+NFATC1-motif No
	PDAC-hypo	117	407	106	418	44	480
	Other	684	7977	874	7787	80	8581

NFKB2-motif: Odds Ratio = 3.35, $P = 8.3\times10^{-23}$

NFATC1-motif: Odds Ratio = 2.26, $P = 2.7\times10^{-11}$

NFKB2+NFATC1-motif: Odds Ratio = 9.83, $P = 3.8\times10^{-24}$

c

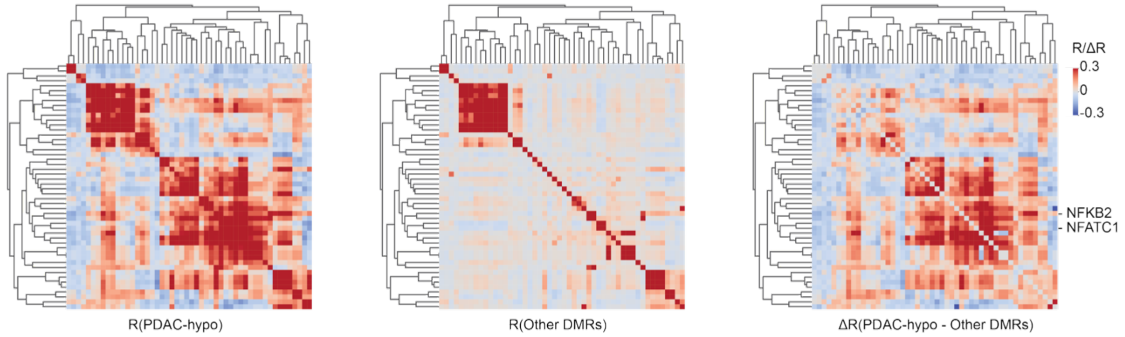

d

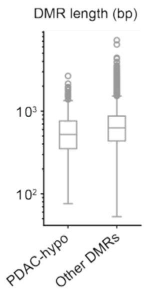

e

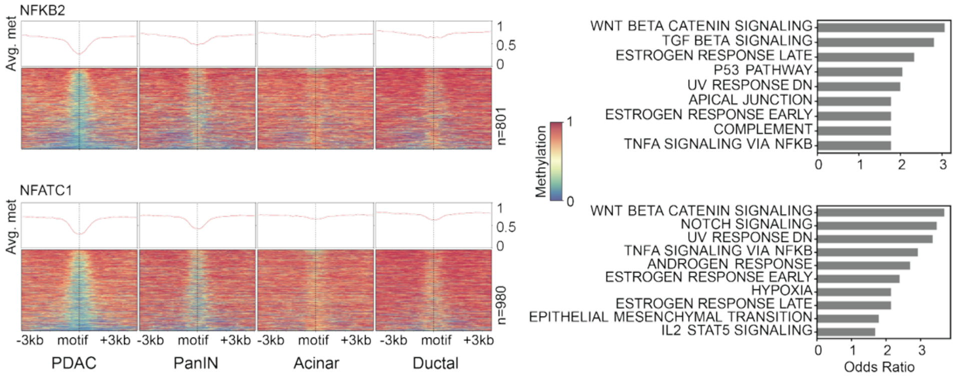

Supplementary Fig. 9: Metilene[3] enables identification of transcription factor co-binding in tumor.

a) Motif enrichment in PDAC-specific DMRs. (top) Top three enriched motifs in DMRs associated with PDAC-specific hypermethylation. No significant enrichment (P<0.001)

was found. (bottom) Top six enriched motifs in DMRs associated with PDAC-specific hypomethylation.

b) Number of DMRs with/without motifs for NFKB2 (left) and NFATC1 (middle), as well as their co-binding (right) in two categories. PDAC-hypo: DMRs (absolute mean difference > 0.5) are hypomethylated in PDAC and hypermethylated in other groups. Other (background): all DMRs (absolute mean difference > 0.5) that are not classified as PDAC-hypo. Two-sided Fisher's exact test is used for calculating the P-value.

c) Pearson correlation coefficient, R, between PDAC-enriched motifs, based on their presence (1) or absence (0) in DMRs. Correlations are shown for PDAC-hypomethylated DMRs (left), other DMRs (middle), and the change in R between these two groups (right).

d) Lengths of PDAC-hypomethylated DMRs (n=524) and other DMRs (n=8661). PDAC-hypomethylated DMRs are slightly shorter than other DMRs. Boxes show the first and third quartiles with the median at the centre and whiskers extend to the minimum and maximum within 1.5x the inter-quartile range.

e) Smoothed methylation of DMRs with NFKB2 motif (top) or NFATC1 motif (bottom), and the gene-sets enriched in these DMRs (right). Of note, the key TFs, NFKB1 (p105/p50; canonical[3]) and NFKB2 (p100/p52; non-canonical[3]), share the core motif[4], limiting motif-only discrimination of their specific effects at metilene[3] DMRs, which is similar for the NFATs[5].

Source data are provided as a Source Data file.

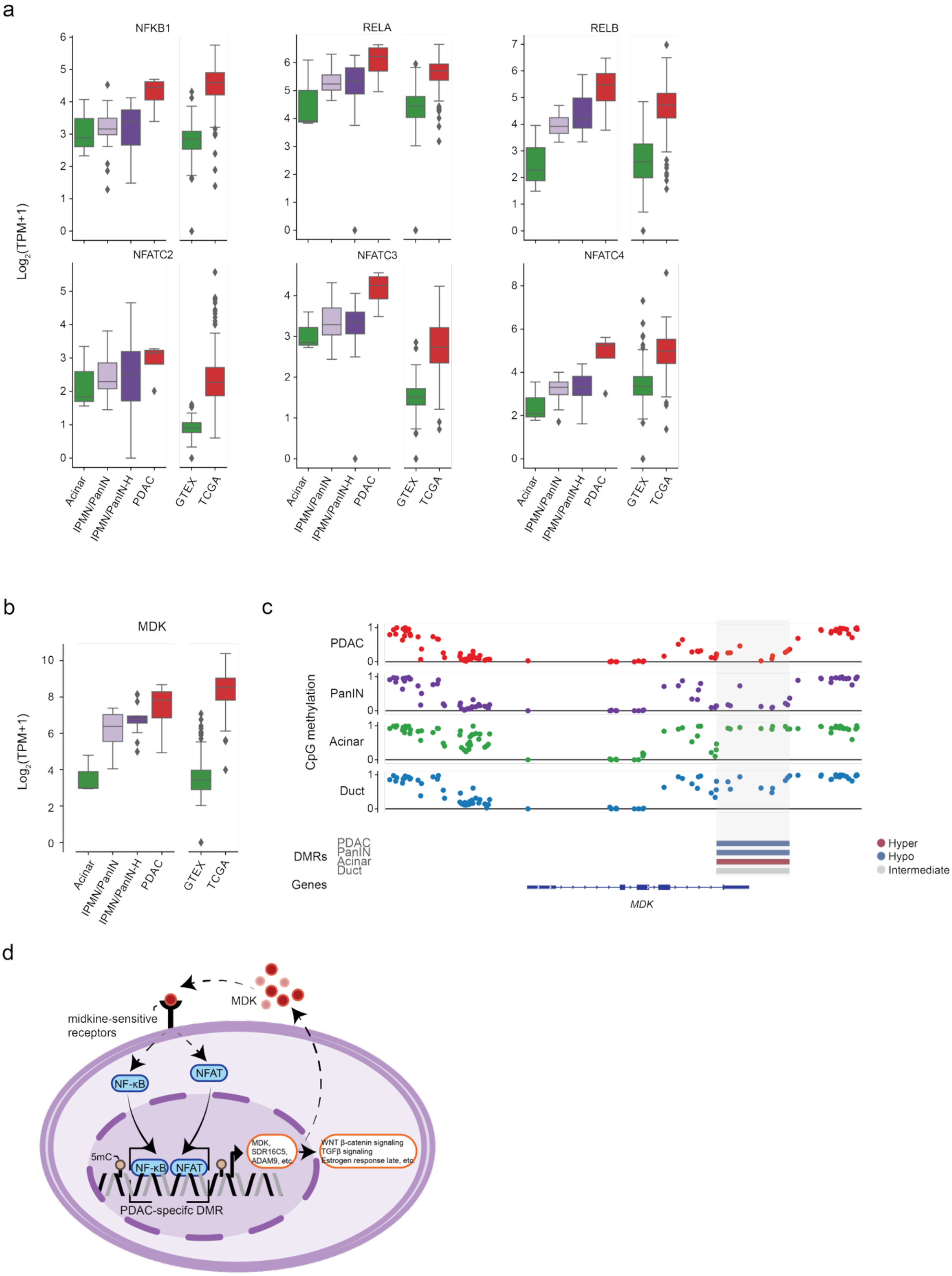

Supplementary Fig. 10: MDK is differentially methylated and expressed between normal and (pre) cancerous samples.

a) Expression of NF-κB family and NFAT family across acinar (n=3), IPMN/PanIN (n=18), high-grade IPMN/PanIN (IPMN/PanIN-H, n=16), PDAC (n=4), GTEx-pancreas (n=167), and TCGA's pancreas adenocarcinoma (PAAD, n=179), from GSE210351[1]

and UCSC Xena[2]. Boxes show the first and third quartiles with the median at the centre and whiskers extend to the minimum and maximum within 1.5x the inter-quartile range.

b) Expression of MDK across acinar (n=3), IPMN/PanIN (n=18), high-grade IPMN/PanIN (IPMN/PanIN-H, n=16), PDAC (n=4), GTEx-pancreas (n=167), and TCGA's pancreas adenocarcinoma (PAAD, n=179), from GSE210351[1] and UCSC Xena[2]. Boxes show the first and third quartiles with the median at the centre and whiskers extend to the minimum and maximum within 1.5x the inter-quartile range.

c) CpG methylation patterns at the MDK gene (chr11:46,400,378-46,406,927). A DMR distinguishes hypomethylated PDAC and PanIN samples from the highly methylated benign tissues. In the left flank region of the DMR, PanIN samples, together with normal samples, exhibit an intermediate methylation pattern, while PDAC samples show a lower methylation pattern.

d) Schematic of a potential regulatory model of how MDK might be involved in PDAC tumor proliferation through a positive feedback loop via NFKB and NFAT.

Source data are provided as a Source Data file.

Reference

1. Liffers, S.T. *et al.* Molecular heterogeneity and commonalities in pancreatic cancer precursors with gastric and intestinal phenotype. *Gut* **72**, 522-534 (2023).
2. Goldman, M.J. *et al.* Visualizing and interpreting cancer genomics data via the Xena platform. *Nature Biotechnology* **38**, 675-678 (2020).
3. Yu, H., Lin, L.B., Zhang, Z.Q., Zhang, H.Y. & Hu, H.B. Targeting NF-κB pathway for the therapy of diseases: mechanism and clinical study. *Signal Transduction and Targeted Therapy* **5**(2020).
4. Chen, F.E., Huang, D.B., Chen, Y.Q. & Ghosh, G. Crystal structure of p50/p65 heterodimer of transcription factor NF-κB bound to DNA. *Nature* **391**, 410-413 (1998).
5. Mognol, G.P., Carneiro, F.R.G., Robbs, B.K., Faget, D.V. & Viola, J.P.B. Cell cycle and apoptosis regulation by NFAT transcription factors: new roles for an old player. *Cell Death & Disease* **7**(2016).
