# Supplementary material for: metilene3: identifying DMRs across multiple conditions with auto-classification
Source: Nat Commun. 2026 Jul 4;17:5848. doi: 10.1038/s41467-026-74931-y (PMC13333024; doi:10.1038/s41467-026-74931-y)
Supplement: Supplementary file 2 — Description of Additional Supplementary Files [file 41467_2026_74931_MOESM2_ESM.pdf]

Description of Additional Supplementary Files

Supplementary Data 1: Simulated DMRs.

Supplementary Data 2: DMRs identified in blood samples.

Supplementary Data 3: DMRs identified in glioma samples.

Supplementary Data 4: DEGs between cluster B and C.

Supplementary Data 5: GSEA on DMRs and DEGs.

Supplementary Data 6: DMRs identified in CSF samples.

Supplementary Data 7: DMRs identified in PDAC samples.

Supplementary Data 8: NFATC1 motif and NFKB2 motif in DMRs.

Supplementary Data 9: DEGs between TCGA-PAAD and matched normal tissues.

Supplementary Software 1: Source code of metilene[3].

Supplementary Software 2: Codes to reproduce figures in this study.
