# Supplementary material for: metilene3: identifying DMRs across multiple conditions with auto-classification
Source: Nat Commun. 2026 Jul 4;17:5848. doi: 10.1038/s41467-026-74931-y (PMC13333024; doi:10.1038/s41467-026-74931-y)
Supplement: Supplementary file 4 — Supplementary Software 1 [file 41467_2026_74931_MOESM4_ESM.zip › metilene3-main/template_unsup.html]

Metilene3 Report


## Metilene3 Report for XXX

### History

Command: XXX

  

Version: XXX

  

Parameters: XXX

  

Start time: XXX

  

End time: XXX

  
  

### Unsupervised

Number of unsupervised DMRs: XXX

  

Number of clusters: XXX

  

Types of DMRs:

  
  
Click to show the Table of clusters

Close  

Clusters of samples:

  
  
Click to show the figures

Close  

PCA:

  
  

Cluster tree:

  
  

Heatmap:

  
  

### Supervised

Number of supervised DMRs: XXX

  

Types of DMRs:

  
  
