# Supplementary material for: metilene3: identifying DMRs across multiple conditions with auto-classification
Source: Nat Commun. 2026 Jul 4;17:5848. doi: 10.1038/s41467-026-74931-y (PMC13333024; doi:10.1038/s41467-026-74931-y)
Supplement: Supplementary file 7 — Transparent Peer Review file [file 41467_2026_74931_MOESM7_ESM.pdf]

nature portfolio

Peer Review File

metilene3: Identifying DMRs Across Multiple Conditions with Auto-Classification

Corresponding Author: Professor Helene Kretzmer

This file contains all reviewer reports in order by version, followed by all author rebuttals in order by version.

Version 0:

Reviewer comments:

Reviewer #1

(Remarks to the Author)
Hoffmann et al. introduced metilene3, an improved version of the metilene, for processing bisulfite sequencing data and identifying differentially methylated regions (DMRs). The critical improvement of this tool appears to be its unsupervised mode of automonously clustering samples without user-provided labels. The authors showed greater confidence in identifying DMRs and highlighted the accuracy of the unsupervised mode in complex contexts of cancer. However, this unsupervised mode is not entirely novel and the validation of metilene3 might require additional and robust evidence. Additionally, the suggested midkine-NFKB-NFAT axis in pancreatic carcinogenesis seems to be overinterpreted. Overall, despite the improved metilene3, the manuscript might not be sufficient for a publication in Nature Communications.

Major issues
1. The identification of MDK was only through computational analysis of DMRs and DEGs but not supported by any experimentally evidence. There are no experimental designs to verify the upregulation of MDK was indeed due to the hypomethylated regions associated with the co-binding of NFKB2 and NFATC1 in pancreatic cancer. The title of the manuscript does not represent overall scope of the manuscript and overclaimed.
2. The authors highlighted the unsupervised mode of metilene3 but this concept of unsupervised clustering in DMR detection is not entirely novel. Several other tools such as Aclust2.0 and MethylScore (using machine learning approach) were developed that can perform similar tasks. How metilene3 outperforms other computational tools might be necessary.
3. In the section of construction of DMTrees, the method that authors used was to assign sample to either one of three categories G, H, and I. Will this categorization be sufficient to keep subtle changes across samples, particularly in the scenario of clustering a large number of samples?
4. In the performance tests on simulated data, the authors showed that metilene3 can identify many more DMRs in both background when compared to either SMART or wgbstools. It should be clarified whether other packages failed to detect critical DMRs. Detection of more number of DMRs does not necessarily mean superior function of metilene3.

(Remarks on code availability)

Reviewer #2

(Remarks to the Author)
I co-reviewed this manuscript with one of the reviewers who provided the listed reports. This is part of the Nature Communications initiative to facilitate training in peer review and to provide appropriate recognition for Early Career Researchers who co-review manuscripts.

(Remarks on code availability)

Reviewer #3

(Remarks to the Author)

DNA methylation is a key epigenetic marker, and comparative analysis of methylation patterns between healthy and diseased samples can yield valuable research insights and potential therapeutic targets. While existing studies have primarily focused on pairwise comparisons under two experimental conditions, the identification and interpretation of differential methylation in complex biological contexts—such as tumor heterogeneity—remain challenging. The authors present metilene3, a computational tool that demonstrates high accuracy in both simulated and real datasets. Notably, the unsupervised mode in metilene3 offers a novel approach for exploring previously unidentified subgroups, thereby expanding its applicability in epigenomic research.

Major :
The authors emphasize that the unsupervised mode represents a key feature of this study; however, the performance evaluation of this mode remains insufficient. Although an analysis of sequencing samples has been conducted, a systematic assessment of the method's accuracy should first be performed using standardized simulated datasets to establish its reliability under controlled conditions.

The comparison based on simulated data requires greater rigor and clarity. The authors claim to apply their method in an unsupervised manner but resort to comparing it with metilene3 in supervised mode, citing that SMART and wgbstools support only supervised approaches. This introduces ambiguity, as the comparison does not fully align with the proposed framework. Given that the primary contribution of this work lies in its unsupervised design, a more compelling validation would involve benchmarking against existing tools under comparable unsupervised settings, where feasible, to ensure a fair and meaningful evaluation.

In the evaluation of DMTree, the authors present hypomethylation events and their associated motifs as supporting evidence. To provide a more comprehensive assessment, hypermethylation events should also be included as additional evidence to strengthen the biological interpretation and balance the analysis.

The authors used two datasets to demonstrate metilene3's accuracy in classifying differential methylation across multiple conditions. Nevertheless, a more systematic analysis of known intra-group variations is necessary to fully illustrate the strengths of the unsupervised model and to highlight the substantial heterogeneity present within the same tumor type.

Minor :
1. Typo: Introduction line 50, should be metilene3 rather than metilene5.
2. The hyperlink for metilene3-doc dose not work, authors should check about it.
3. With regard to the code, a comprehensive example should be provided, including parameters such as -refs and -anno, to clearly define the required input format.
4. Figure 5C should include a legend to indicate the meaning of each colored dot clearly.

(Remarks on code availability)

Reviewer #4

(Remarks to the Author)
In this manuscript, Zhu et al. present Metilene3, a promising new tool for DNA methylation analysis. The method combines unsupervised DMR detection with DMTree-based sample clustering, making it particularly useful in settings where predefined labels are difficult to assign. The authors also highlight methylation changes associated with an NFKB2–NFATC1 regulatory motif in pancreatic cancer, suggesting that the tool has the potential to reveal novel biological insights. At the same time, several points would benefit from additional clarification or validation to further strengthen the utility of the method. The specific concerns are outlined below. Addressing these issues would help more clearly demonstrate the advantages and practical value of Metilene3.

Major Points

1. Limited Benchmarking Against Alternative Unsupervised Approaches
Although the authors highlight the limitations of traditional unsupervised DNA methylation analyses that rely on selecting highly variable CpGs, the manuscript does not provide quantitative comparisons with simpler approaches in the context of unsupervised clustering. Without baseline analyses such as clustering based on top variable CpGs or principal components, it remains difficult to determine how much improvement DMTree offers over existing methods. The paper qualitatively shows that PCA using all CpGs fails to separate certain groups while DMR based features succeed, but this alone is not sufficient to establish a clear methodological advantage. Including a basic benchmark using commonly used unsupervised methods or an equivalent number of highly variable CpGs would help more convincingly demonstrate the added value of metilene3 and further strengthen the overall assessment of the method.

2. Limited Quantitative Support for Clustering Evaluation
The evaluation of the clustering results in the manuscript is primarily qualitative for the real datasets, based on visual inspection and alignment with known biological categories. While the findings are compelling, the absence of quantitative validation leaves uncertainty regarding the statistical robustness and reproducibility of the DMTree-based classification. To improve the rigor of the analysis, the authors may wish to include some form of quantitative assessment of clustering performance and stability, leaving the specific approach to their discretion. This would help to more objectively support the

biological interpretations drawn from the clustering results.

3. Limited Evaluation on Low-Purity and Heterogeneous Samples
The study does not adequately assess performance on low tumor purity or heterogeneous samples, which are common in clinical settings. Although a small cfDNA dataset (5 vs 4 samples) is used as a low-purity test case, this single example is limited in scope. There is no systematic benchmarking across known purity gradients (e.g., DNA mixing experiments) to assess detection limits. Notably, a glioblastoma sample (AK015) clustered with normal tissues, possibly due to low tumor content, highlighting potential misclassification risks. Additional evaluation on diverse or synthetic low-purity datasets would help clarify the tool's reliability under clinically relevant conditions.

4. Gaps in Algorithm Design and Handling of Heterogeneity
The DMTree relies on a greedy, binary splitting strategy driven by the most "impactful" DMRs. This approach may overlook moderate but biologically relevant methylation patterns and is susceptible to suboptimal splits if large differences arise from technical noise or batch effects. In addition, the method does not model continuous gradients or admixture, forcing samples into discrete branches. Such a design may be insufficient for highly heterogeneous tumors, where methylation changes often reflect continuous evolutionary processes rather than distinct groups. The manuscript does not address how DMTree performs under these conditions or how covariates such as tumor purity or batch effects should be handled, leaving uncertainty about its robustness in real-world applications. While extending the methodology would be ideal, if this is not feasible within the current revision, the authors should at least acknowledge these limitations and outline potential directions for future improvements in the Discussion section.

5. Computational Predictions Require Functional Validation
The manuscript presents compelling biological hypotheses based on computational findings. However, some interpretations appear to go beyond what the available data can support. In the pancreatic cancer analysis, the authors identify DMRs enriched for NF-κB and NFAT motifs and propose a novel signaling axis involving midkine (MDK), potentially driven by a feedback loop between these transcription factors. While the hypothesis is intriguing and biologically plausible, it is based solely on motif enrichment and gene expression correlations, without experimental validation or prior literature directly supporting the proposed mechanism. In vivo or in vitro experiments would be needed to substantiate these claims and demonstrate the tool's utility in driving biological discovery.

6. Potential for Clinical Insight With Additional Outcome-Based Analyses
Metilene3 successfully stratifies samples based on methylation patterns, such as separating normal and tumor tissues and identifying subgroups within IDH-mutant gliomas, and the method is technically promising. However, the manuscript does not sufficiently demonstrate how these findings translate into clinical benefit. For example, the newly identified subclusters in IDH-mutant glioblastoma (Cluster B and Cluster C) show differences in EMT-related pathways, but their associations with patient outcomes, treatment responses, or other clinical indicators have not been examined. Additional analyses exploring potential relationships with available clinical parameters would be valuable.

Minor Points

1. Category "I" represents samples that cannot be clearly assigned to groups G or H, but the main text gives little explanation of how these intermediate cases are handled during DMTree construction (e.g. lines 103-104). It is unclear whether they influence clustering or are treated largely as noise. Although the Methods describe a procedure, the narrative is insufficient, and additional clarification in the main text would be helpful.

2. The paper does not describe how key parameters were set. For example, the criteria for finalizing clusters in the DMTree are mentioned, but the threshold values and their rationale are not provided. It is also unclear what significance or effect size thresholds were used for calling DMRs, as no explicit FDR or p-value cutoff is stated in the main text. Clear reporting of these details would be helpful.

3. Metilene3 reports a very large number of DMRs in several analyses, which raises minor questions about how many of these regions have meaningful effect sizes or biological relevance. Aside from post-hoc motif analyses, the manuscript does not describe how important DMRs are prioritized. A brief explanation of how these large DMR sets should be interpreted or filtered would be helpful.

4. The examples of "double-switch" DMR patterns at the TRAK1 and TXNRD1 loci provide key evidence for the biological insights enabled by metilene3's local clustering approach (lines 318-338). Given their importance, it may be worth considering moving at least one representative example into the main figures to help readers appreciate the significance of these findings without relying solely on the Extended Data.

5. Some terms and acronyms appear before being defined (for example, "MDS" for methylation distance score), which may confuse readers on first encounter.

(Remarks on code availability)
I was not able to access the source code, so I could not assess whether it includes sufficient documentation (such as a README), installation instructions, or runnable scripts. Without access to the code, it is not possible to evaluate the reproducibility of the analyses or determine whether the code is a useful resource for the community. I recommend that the authors make the code publicly available to support transparency and reproducibility.

Version 1:

Reviewer comments:

Reviewer #1

(Remarks to the Author)
While the authors have addressed the major issues related to the novelty of Metilene3 and its improved performance, the revised manuscript still does not provide new biological insights resulting from the use of this tool. With the claims regarding biological interpretation toned down, it remains unclear what new biological insights this study ultimately provides.

(Remarks on code availability)

Reviewer #2

(Remarks to the Author)
I co-reviewed this manuscript with one of the reviewers who provided the listed reports. This is part of the Nature Communications initiative to facilitate training in peer review and to provide appropriate recognition for Early Career Researchers who co-review manuscripts.

(Remarks on code availability)

Reviewer #3

(Remarks to the Author)
The authors have addressed all my previous concerns. However, the benchmarking data should be made available in the GitHub repository or the supplementary materials to ensure research reproducibility and facilitate comparison with other studies.

(Remarks on code availability)

Reviewer #4

(Remarks to the Author)
The revised manuscript has addressed most of my previous comments in a careful and thoughtful manner, and I appreciate the authors' efforts to improve the study. I have only two remaining points that, if clarified, would further strengthen the manuscript:

Regarding Major Point 3:
The additional simulations and DNA-mixing analyses are appreciated. However, it remains unclear how these results translate to real-world datasets. Could the authors provide a quantitative estimate of the minimal tumor purity required for reliable clustering, and clarify how performance degrades below this threshold?

Regarding Major Point 4:
The clarification of the DMTree framework is helpful. However, the basis for its improved robustness remains unclear. Could the authors clarify whether the performance gain is primarily driven by the use of DMR-level aggregation rather than the tree-based strategy itself?

(Remarks on code availability)

Version 2:

Reviewer comments:

Reviewer #4

(Remarks to the Author)
All of my concerns have been satisfactorily addressed in this revised version of the manuscript, as well as in the authors' detailed responses.

(Remarks on code availability)

Open Access This Peer Review File is licensed under a Creative Commons Attribution 4.0 International License, which permits use, sharing, adaptation, distribution and reproduction in any medium or format, as long as you give appropriate credit to the original author(s) and the source, provide a link to the Creative Commons license, and indicate if changes were made.
In cases where reviewers are anonymous, credit should be given to 'Anonymous Referee' and the source.
The images or other third party material in this Peer Review File are included in the article’s Creative Commons license, unless indicated otherwise in a credit line to the material. If material is not included in the article’s Creative Commons license and your intended use is not permitted by statutory regulation or exceeds the permitted use, you will need to obtain permission directly from the copyright holder.
To view a copy of this license, visit https://creativecommons.org/licenses/by/4.0/

We thank you for your constructive feedback and appreciate the detailed suggestions for improvement and missing analyses. We took your concerns very seriously and adjusted the manuscript strongly, in particular, the following three key concerns:

1. We improved the benchmarking on simulated data by
   - adding two new tools to the benchmarking
   - performing a systematic, controlled benchmark for the unsupervised mode (new Fig. 3).
   - simulating more complex data, including batch effects, purity mixtures and group heterogeneity.
   - validating the unsupervised mode against standard unsupervised methods, i.e., hierarchical and k-means clustering.
   - adding a robustness and stability analysis.

2. We rewrote several parts of the manuscript and balanced the biological claims as well as the overall tone and positioned our tool more precisely in the field.

3. Improved accessibility to the code and the methods sections.

We believe these revisions strengthen the manuscript and address the concerns that were raised.

Please find our detailed point-by-point response below.

Reviewer #1 (Remarks to the Author):
Hoffmann et al. introduced metilene3, an improved version of the metilene, for processing bisulfite sequencing data and identifying differentially methylated regions (DMRs). The critical improvement of this tool appears to be its unsupervised mode of automonously clustering samples without user-provided labels. The authors showed greater confidence in identifying DMRs and highlighted the accuracy of the unsupervised mode in complex contexts of cancer. However, this unsupervised mode is not entirely novel and the validation of metilene3 might require additional and robust evidence. Additionally, the suggested midkine-NFKB-NFAT axis in pancreatic carcinogenesis seems to be overinterpreted. Overall, despite the improved metilene3, the manuscript might not be sufficient for a publication in Nature Communications.

Major issues
1. The identification of MDK was only through computational analysis of DMRs and DEGs but not supported by any experimentally evidence. There are no experimental designs to verify the upregulation of MDK was indeed due to the hypomethylated regions associated with the co-binding of NFKB2 and NFATC1 in pancreatic cancer. The title of the manuscript does not represent overall scope of the manuscript and overclaimed.

Response: We thank the reviewer for this very reasonable comment. We fully agree that we don't provide any validation and should tone down these parts, as well as the title. Unfortunately, our DNA methylation and transcriptomic data are from different studies and therefore don't match by sample, which leaves us with co-occurrence rather than direct correlation.

Action taken: We propose an adjusted and more technical title to better reflect the manuscript's scope. In addition, we toned down the MDK section and clarified that our method provides evidence and correlations that are useful for hypothesis generation but still require experimental validation.

2. The authors highlighted the unsupervised mode of metilene3 but this concept of unsupervised clustering in DMR detection is not entirely novel. Several other tools such as Aclust2.0 and MethylScore (using machine learning approach) were developed that can perform similar tasks. How metilene3 outperforms other computational tools might be necessary.

Response: We appreciate this pointer and apologize for having overlooked Aclust2.0 and MethylScore in our initial related-work survey; both were surprisingly hard to find via a Google search.
We have now acknowledged them in the manuscript. While Aclust2.0 targets array data only, we added MethylScore to our benchmarking (although it was difficult to install, requiring a combination of GitHub and Nextflow/Docker for different parts of the processing). Furthermore, MethylScore is specifically designed for plant WGBS, which methylate more

sequence contexts than mammals and show different methylation distributions. Since MethylScore detects DMRs between any combinations of samples without a search for robust patterns, it appears very sensitive to the simulated noise and batch effects.
While parts of this approach are similar to the idea behind unsupervised DMR calling, we would like to highlight that metilene3's clustering of samples via DMTree is a crucial part of the unsupervised mode. This step facilitates the focus on stable, recurrent sample groupings, which are subsequently used for the supervised DMR calling, resulting in groups and DMRs that are less prone to noise or batch effects. Therefore, we would like to emphasize the novelty of metilene3, which consists of a highly sensitive, unsupervised DMR calling, followed by a new clustering routing which functions as a self-correction step to focus on recurrent groupings, followed by the final DMR calling using the previously de novo sample groupings.

Action taken: We included MethylScore in the benchmarking (**Fig. 2**, **ED 2**). Unfortunately, and not surprisingly given the by-sample segmentation approach, MethylScore does not faithfully recover DMRs, and it also overpredicts or underpredicts their length. We adjusted the text to better highlight the novelty of metilene3.

3. In the section of construction of DMTrees, the method that authors used was to assign sample to either one of three categories G, H, and I. Will this categorization be sufficient to keep subtle changes across samples, particularly in the scenario of clustering a large number of samples?

Response: We appreciate this question. In metilene3, G/H/I is a local, per-DMR assignment into methylated or unmethylated states with uncertainty (I), derived from continuous methylation distances; it is not a one-shot discretization of all samples overall. CpG methylation at many loci is near-bimodal, and methylation rates generally cluster around 0, 1, or to a lesser extent 0.5, so category "I" reflects an ambiguous/intermediate state when the distances to G and H are similar. The DMTree aggregates evidence across many DMRs, so fine-grained signals accumulate over splits; "I" cases at one split are often assigned to G or H samples in other DMRs. Our preliminary assignment, followed by DMTree to define stable groups, allows us to find DMR patterns in larger groups, where the complexity of samples~categories~DMRs would otherwise make it difficult to detect robust patterns.

Action taken: We have added a better description of category I and its biological reasoning to the manuscript. It now states that G/H/I are preliminary, local labels that improve noise robustness while preserving recurrent, stable differences via aggregated evidence (DMTree). Additionally, we now report the mean methylation for G/H/I per DMR and the per-sample DMR methylation values in a separate file.

4. In the performance tests on simulated data, the authors showed that metilene3 can identify many more DMRs in both background when compared to either SMART or wgbstools. It should be clarified whether other packages failed to detect critical DMRs. Detection of more number of DMRs does not necessarily mean superior function of metilene3.

Response: We would like to apologize for not having stated this statistic clearly in the figures and agree that sensitivity alone is insufficient. However, we would like to highlight that we explicitly stated in the manuscript that "*Despite the clear differences in sensitivity between methods, all DMR callers, including metilene3, exhibit false positive rates smaller than 1%.*" and that "*The overall accuracy reaches 0.885 in background 1 and 0.860 in background 2.*".

Action taken: To better highlight this point and provide a more quantitative metric, we added the Jaccard index as a benchmarking metric to measure the similarity between detected and simulated DMRs (**Fig. 2c**), showing that metilene3 achieved a higher Jaccard index compared to other tools. In addition, we have added a panel (**Fig. 2d**) showing the numbers of true positives (simulated DMRs detected), false positives (regions incorrectly detected), and false negatives (missed DMRs) in relation to the length of the respective DMR.

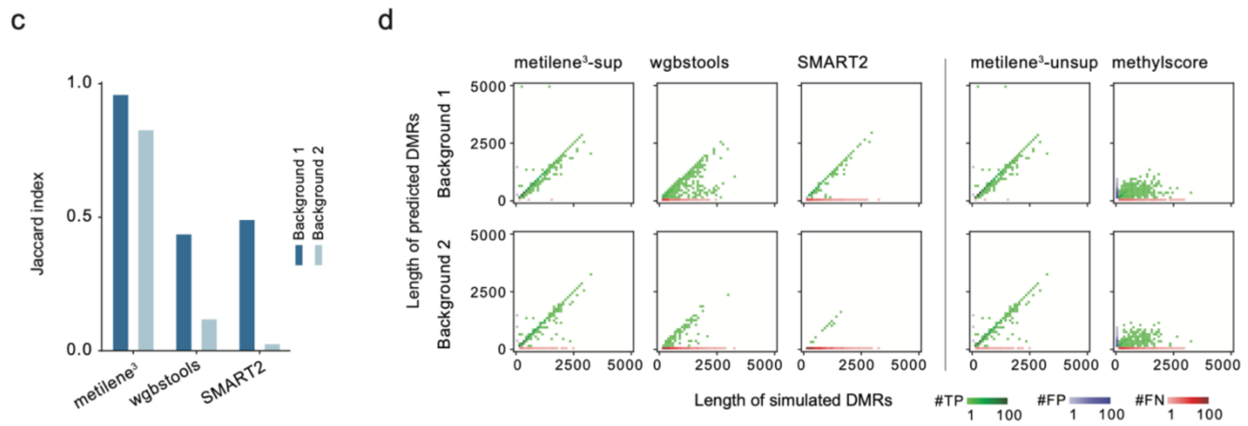

Reviewer #2 (Remarks to the Author):
I co-reviewed this manuscript with one of the reviewers who provided the listed reports. This is part of the Nature Communications initiative to facilitate training in peer review and to provide appropriate recognition for Early Career Researchers who co-review manuscripts.

Response: We thank reviewer #2 for their effort and time in helping us improve the manuscript through their co-review.

Reviewer #3 (Remarks to the Author):

DNA methylation is a key epigenetic marker, and comparative analysis of methylation patterns between healthy and diseased samples can yield valuable research insights and potential therapeutic targets. While existing studies have primarily focused on pairwise comparisons under two experimental conditions, the identification and interpretation of differential methylation in complex biological contexts—such as tumor heterogeneity—remain challenging. The authors present metilene3, a computational tool that demonstrates high accuracy in both simulated and real datasets. Notably, the unsupervised mode in metilene3 offers a novel approach for exploring previously unidentified subgroups, thereby expanding its applicability in epigenomic research.

Major:

The authors emphasize that the unsupervised mode represents a key feature of this study; however, the performance evaluation of this mode remains insufficient. Although an analysis of sequencing samples has been conducted, a systematic assessment of the method's accuracy should first be performed using standardized simulated datasets to establish its reliability under controlled conditions.

Response: We thank the reviewer for pointing this out. We agree that we focused mainly on the supervised multi-group benchmark, and presented the results of the unsupervised mode only very indirectly. We now added a systematic, controlled benchmark specifically for the unsupervised mode.

Action taken: We added a **new Figure 3** that focuses solely on benchmarking the unsupervised mode. Additionally, we re-designed the simulated data to also include sample purity/heterogeneity as well as batch effects. Specifically, we

1. Introduce simulated purities, batch effects, and two confounding factors to increase the heterogeneity within the groups (**ED Fig. 2**).
2. Benchmark the unsupervised mode (DMTree) against common clustering methods used in DNA methylation analysis, including hierarchical clustering (HC) and K-means clustering (**Fig. 3**).
3. We performed a subsampling experiment (40 random samples, varying size of G1) and benchmarked the clustering performance of all three methods across the selected group/cluster size parameters. To this end, we assessed robustness with respect to group size, i.e., the proportion of G1 (5-25% of samples) and the remaining samples from G2-G5.

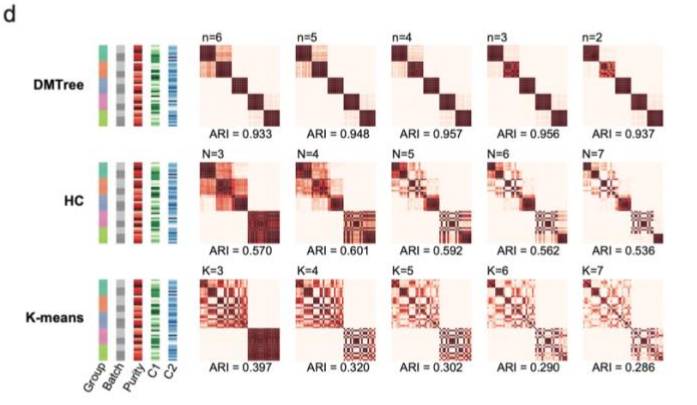

Fig. 3 d: Consensus matrix across 50 resampled runs. Samples are ordered by simulated groups, batches and purity. ARI = average rand index.

4. The Rand index is used to measure the accuracy of the subsampling experiments across different types of ‘cluster number’ parameters (n = minimum group size for metilene[3], N = # groups in the hierarchical clustering, k = #clusters in K-means clustering). ARI = average rand index.
5. Specifically, DMTree identifies smaller groups more reliably than classic approaches such as hierarchical clustering or PCA with K-means. Recoverability refers to the correct grouping of samples in G1, i.e., not with their most similar samples, which are from G2. Of note, for each clustering, the ‘cluster number’ associated parameter is varied across scales to show its effect (**Fig. 3d and e**).

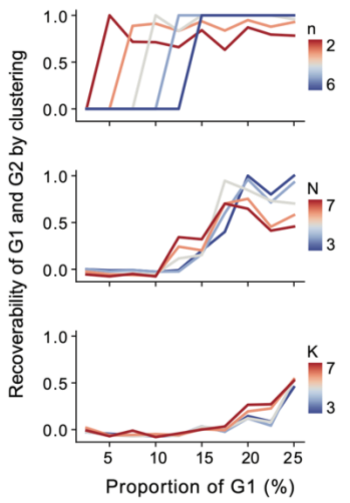

The comparison based on simulated data requires greater rigor and clarity. The authors claim to apply their method in an unsupervised manner but resort to comparing it with metilene3 in supervised mode, citing that SMART and wgbstools support only supervised approaches. This introduces ambiguity, as the comparison does not fully align with the proposed framework. Given that the primary contribution of this work lies in its unsupervised design, a more compelling validation would involve benchmarking against existing tools under comparable unsupervised settings, where feasible, to ensure a fair and meaningful evaluation.

Response: The reviewer highlights an important point, which is the very limited of benchmarking for the unsupervised mode using simulated data and comparing it to other tools. Despite searching the literature, we didn’t find other algorithms with this ability. However, thanks to reviewer #1, we are now aware of Aclust2.0 and MethylScore. While Aclust2.0 is designed for methylation array data (which is not comparable), and MethylScore is specifically for plants. However, we still used the opportunity to benchmark against MethylScore. Unfortunately, MethylScore was challenging to install, since some of the functions were only available via the GitHub version, and another part required a Docker-based installation. Nevertheless, we stitched these two distributions together to cover the entire workflow. Another limitation of MethylScore is that it does not provide global sample clustering (only locally, meaning groupings), so hierarchical/lineage relationships cannot be inferred.

Action taken: In addition to the new analysis introduced above, we added a benchmarking section comparing it with MethylScore (**Fig. 2**, **ED2**). To deconvolute the unsupervised and supervised sections, we clearly separated them in the figures (**Fig. 2 supervised and new Fig. 3 unsupervised**) and manuscript.

In the evaluation of DMTree, the authors present hypomethylation events and their associated motifs as supporting evidence. To provide a more comprehensive assessment, hypermethylation events should also be included as additional evidence to strengthen the biological interpretation and balance the analysis.

Response: The reviewer highlights an important focus in our analysis and agrees that a more balanced reporting would strengthen our analysis. While we always refer to hypomethylation, we do not explicitly state that this means that other samples are hypermethylated. Therefore, in Figure 3, we state that C/EBPb TFBS are "hypomethylated in myeloid cells" and that this is relevant to protect lymphoid cells from transdifferentiation, which rather refers to hypermethylation in the lymphoid lineage.
Nevertheless, our original PDAC TFBS analysis contrasted hypo-DMRs with all DMRs, and we followed up on this, given the strong enrichment of relevant TFs. The reviewer is absolutely right to highlight that we should also show PDAC-hyper, even though no clear enrichment was detected.

Action taken: We calculated the enrichment in PDAC-specific hyper DMRs and added this information to the manuscript and the **ED Fig. 9a**. However, no significantly enriched motifs were found.

The authors used two datasets to demonstrate metilene3's accuracy in classifying differential methylation across multiple conditions. Nevertheless, a more systematic analysis of known intra-group variations is necessary to fully illustrate the strengths of the unsupervised model and to highlight the substantial heterogeneity present within the same tumor type.

Response: We thank the reviewer for this suggestion and are happy to introduce greater within-group variance to improve our benchmarking. For our **new Fig. 3**, which focuses on benchmarking the unsupervised mode, we simulated datasets with increasing complexity.

Action taken: We specifically designed experiments to also include sample purity, group heterogeneity, confounding effects, and batch effects. Please see the response to your first concern.

Minor:
1. Typo: Introduction line 50, should be metilene3 rather than metilene5.
2. The hyperlink for metilene3-doc does not work, authors should check about it.
3. With regard to the code, a comprehensive example should be provided, including parameters such as -refs and -anno, to clearly define the required input format.
4. Figure 5C should include a legend to indicate the meaning of each colored dot clearly.

Action taken:

- We would like to apologize for the confusion: the superscript 5 refers to the citation of the previous version of metilene. We have adjusted the citation to not be confused the new version.
- We are not sure why the hyperlink does not work. Even in private browsing mode, it still works for us. We have included it here (https://zzhu1372.github.io/metilene3-doc) and provided a pdf as reviewer material (metilene3-doc-pdf.zip). Additionally, all code is available on GitHub and attached here as reviewer material (metilene3-code.zip and metilene3-fig-code.zip).
- We apologize for this – the detailed example is provided within the link above and can now also be found in the reviewer material.
- We have added the legend to the PCA.

Reviewer #4 (Remarks to the Author):

In this manuscript, Zhu et al. present Metilene3, a promising new tool for DNA methylation analysis. The method combines unsupervised DMR detection with DMTree-based sample clustering, making it particularly useful in settings where predefined labels are difficult to assign. The authors also highlight methylation changes associated with an NFKB2–NFATC1 regulatory motif in pancreatic cancer, suggesting that the tool has the potential to reveal novel biological insights. At the same time, several points would benefit from additional clarification or validation to further strengthen the utility of the method. The specific concerns are outlined below. Addressing these issues would help more clearly demonstrate the advantages and practical value of Metilene3.

Major Points

1. Limited Benchmarking Against Alternative Unsupervised Approaches

Although the authors highlight the limitations of traditional unsupervised DNA methylation analyses that rely on selecting highly variable CpGs, the manuscript does not provide quantitative comparisons with simpler approaches in the context of unsupervised clustering. Without baseline analyses such as clustering based on top variable CpGs or principal components, it remains difficult to determine how much improvement DMTree offers over existing methods. The paper qualitatively shows that PCA using all CpGs fails to separate certain groups while DMR based features succeed, but this alone is not sufficient to establish a clear methodological advantage. Including a basic benchmark using commonly used unsupervised methods or an equivalent number of highly variable CpGs would help more convincingly demonstrate the added value of metilene3 and further strengthen the overall assessment of the method.

Response: We agree with the reviewer's comment and have added additional comparisons to more traditional unsupervised DNA methylation analyses. This was extremely helpful, as we discovered a strong batch effect in the PDAC dataset that was only present when

focusing on the most variable CpGs. This led us to simulate batch effects and benchmark our method under such conditions.

Action taken: In a **new Fig. 3**, we added hierarchical clustering and PCA analyses with k-means clustering to all datasets, using the top variable CpGs. We further added new simulations, including batch effects and confounders as well as purity levels, showing that the local clustering, together with the 'recurrent DMR pattern' approach in metilene3, robustly overcomes batch effects, as detected by PCA and hierarchical clustering. Additionally, we now run the PCA analyses using all CpGs, the top 1% most variable, the top x most variable (x equal to the number of CpGs used for DMTree), e.g., as shown in **Fig. 6c,** as well as **ED Fig. 3, 5b, 6a**.

2. Limited Quantitative Support for Clustering Evaluation
The evaluation of the clustering results in the manuscript is primarily qualitative for the real datasets, based on visual inspection and alignment with known biological categories. While the findings are compelling, the absence of quantitative validation leaves uncertainty regarding the statistical robustness and reproducibility of the DMTree-based classification. To improve the rigor of the analysis, the authors may wish to include some form of quantitative assessment of clustering performance and stability, leaving the specific approach to their discretion. This would help to more objectively support the biological interpretations drawn from the clustering results.

Response: We thank the reviewer for pointing this out. We focused mainly on the novel, supervised multi-group benchmark, and presented the results of the unsupervised mode only very indirectly. Nevertheless, we agree and have added a systematic, controlled benchmark specifically for the unsupervised mode.

Action taken: We added a **new Figure 3** that focuses on benchmarking of robust and reproducible clustering performance of metilene3 using the Rand index as a quantitative metric. The benchmarking is contrasted with classical methods, namely hierarchical and k-means clustering. Specifically, we

1. Introduce simulated purities, batch effects, and two confounding factors to increase the heterogeneity within the groups (**ED Fig. 2**).
2. Benchmark the unsupervised mode (DMTree) against common clustering methods used in DNA methylation analysis, including hierarchical clustering (HC) and K-means clustering (**Fig. 3**).
3. We performed a subsampling experiment (40 random samples, varying size of G1) and benchmarked the clustering performance and robustness of all three methods across the selected group/cluster size parameters. To this end, we assessed robustness with respect to group size, i.e., the proportion of G1 (5-25% of samples) and the remaining samples from G2-G5.
4. The Rand index is used to measure the accuracy of the subsampling experiments across different types of 'cluster number' parameters (n = minimum group size for

metilene[3], N = # groups in the hierarchical clustering, k = #clusters in K-means clustering). ARI = average rand index.

5. Specifically, DMTree identifies smaller groups more reliably than classic approaches such as hierarchical clustering or PCA with K-means. Recoverability refers to the correct grouping of samples in G1, i.e., not with their most similar samples, which are from G2. Of note, for each clustering, the 'cluster number' associated parameter is varied across scales to show its effect (**Fig. 3d and e**).

3. Limited Evaluation on Low-Purity and Heterogeneous Samples

The study does not adequately assess performance on low tumor purity or heterogeneous samples, which are common in clinical settings. Although a small cfDNA dataset (5 vs 4 samples) is used as a low-purity test case, this single example is limited in scope. There is no systematic benchmarking across known purity gradients (e.g., DNA mixing experiments) to assess detection limits. Notably, a glioblastoma sample (AK015) clustered with normal tissues, possibly due to low tumor content, highlighting potential misclassification risks. Additional evaluation on diverse or synthetic low-purity datasets would help clarify the tool's reliability under clinically relevant conditions.

Response: We agree that clinical reliability requires explicit tests of purity, admixture, and heterogeneity and that we could have better presented our analysis. We expanded and adjusted our benchmarking strategy according to the reviewers suggestions.

Action taken: In the simulations, we have a 'mixing factor' affecting the delta between samples. We have now also added a DNA-mixing experiment, designated as a 'purity factor' affecting the fraction of simulated groups (G1-G5) and a control group (Ctr.), see **Fig. 2**. Nevertheless, we have added stronger admixture and more heterogeneous data to better reflect on this in **Fig. 3**. Please see the response above.

Response: Regarding the AK015, given its transcriptomic profile, specifically with comparable TNC expression, we speculate that it might not be as unpure as the methylation profiles suggest but rather differs in proliferation and methylation-wise from the others. In addition, specifically for the IDH-mutant specific DMRs, the sample showed a rather strong deviation from the other normals and a tendency towards cancer, while other cancer-specific regions seem to really reflect normal samples. Thus the effect was not equally distributed across DMRs, making purity-issues a less likely cause. However, even if it was of low purity, this sample was not flagged in the original publication and should have, in that case, been considered as low quality. Therefore, metilene3 seemed useful for flagging the sample and allowing further research into the reason behind this.

Action taken: In addition to the analysis stated above, we performed PCA on the transcriptomic data for AK015 and showed that it clusters with the other cancer samples, though with a tendency toward the controls. We added this information to the **ED Fig. 6c** and

adjusted the manuscript, including a brief discussion of the cause of the deviation of this specific sample.

4. Gaps in Algorithm Design and Handling of Heterogeneity

The DMTree relies on a greedy, binary splitting strategy driven by the most “impactful” DMRs. This approach may overlook moderate but biologically relevant methylation patterns and is susceptible to suboptimal splits if large differences arise from technical noise or batch effects. In addition, the method does not model continuous gradients or admixture, forcing samples into discrete branches. Such a design may be insufficient for highly heterogeneous tumors, where methylation changes often reflect continuous evolutionary processes rather than distinct groups. The manuscript does not address how DMTree performs under these conditions or how covariates such as tumor purity or batch effects should be handled, leaving uncertainty about its robustness in real-world applications. While extending the methodology would be ideal, if this is not feasible within the current revision, the authors should at least acknowledge these limitations and outline potential directions for future improvements in the Discussion section.

Response: We appreciate this important point. In DMTree the split at each node is driven by many DMR-level votes (local G/H/I assignments), not by a single hard threshold, and then evidence is aggregated across DMRs along the tree. This design helps to capture effects that are individually potentially moderate but consistent, and thereby helps to overcome batch effects and purity issues. That said, we agree that a binary tree does not explicitly model continuous gradients, however, since it is supposed to represent an (explainable) clustering, completely continuous dynamics will not be captured in full detail, but rather split into multiple groups (e.g., like in CLL where a continuous cell-of-origin landscape is split into three groups https://www.nature.com/articles/ng.3488). To this end, we would like to highlight that a developmental trajectory of HSCs into the other blood cells is represented in Fig. 4. While the general lineage history is well presented, it of course does not show a fully continuous trajectory.

Action taken: To better illustrate these points, we

(i) Quantify DMTree’s behavior under admixture, batch perturbations, and group-size imbalance, which we now show in a new **Fig. 3** and the adjusted text.
(ii) Provide DMR and sample-specific methylation outputs.
(iii) Add a Kruskal-Wallis test for all DMRs to better capture DMRs that show stronger within-group variance and flag these as potentially caused by batch/admixture effects.
(iv) Acknowledge in the discussion that very continuous developments and groups will be split by the tree and will not necessarily be reflected as a very continuous clustering.

5. Computational Predictions Require Functional Validation
The manuscript presents compelling biological hypotheses based on computational findings. However, some interpretations appear to go beyond what the available data can support. In the pancreatic cancer analysis, the authors identify DMRs enriched for NF-κB and NFAT motifs and propose a novel signaling axis involving midkine (MDK), potentially driven by a feedback loop between these transcription factors. While the hypothesis is intriguing and biologically plausible, it is based solely on motif enrichment and gene expression correlations, without experimental validation or prior literature directly supporting the proposed mechanism. In vivo or in vitro experiments would be needed to substantiate these claims and demonstrate the tool's utility in driving biological discovery.

Response: We thank the reviewer for this very reasonable comment. We fully agree that we don't provide any validation and should have been less strong in stating these findings. Overall, we would like to highlight that, like any other DMR caller, metilene3's main utility is to design and build hypotheses, and it cannot, by any means, provide evidence for any biological discovery. We apologize for using overly strong phrasing here.

Action taken: We propose an adjusted and more technical title to better reflect the manuscript's scope. In addition, we toned down the MDK section and clarified that our method provides evidence and correlations that are useful for hypothesis generation but still require experimental validation.

6. Potential for Clinical Insight With Additional Outcome-Based Analyses
Metilene3 successfully stratifies samples based on methylation patterns, such as separating normal and tumor tissues and identifying subgroups within IDH-mutant gliomas, and the method is technically promising. However, the manuscript does not sufficiently demonstrate how these findings translate into clinical benefit. For example, the newly identified subclusters in IDH-mutant glioblastoma (Cluster B and Cluster C) show differences in EMT-related pathways, but their associations with patient outcomes, treatment responses, or other clinical indicators have not been examined. Additional analyses exploring potential relationships with available clinical parameters would be valuable.

Response: We thank the reviewer for their supportive general evaluation. We agree that the translation into clinics was not immediately apparent. We used RNA-seq phenotyping to show that there are clinically relevant transcriptomic differences between these groups that would not be visible without the methylation-based distinction. However, due to the very limited number of additional clinical parameters available for the patients, we were unable to present other associations.

Action taken: We now stratified these two smaller groups by overall progression-free survival (PFS). While the sample sizes are unfortunately very small, we visually found differences between cluster B and C. However, due to an outlier this result seems extremely case dependent. We therefore have included this analysis here as a reviewer-only figure, since there is too little evidence to support an actual claim.

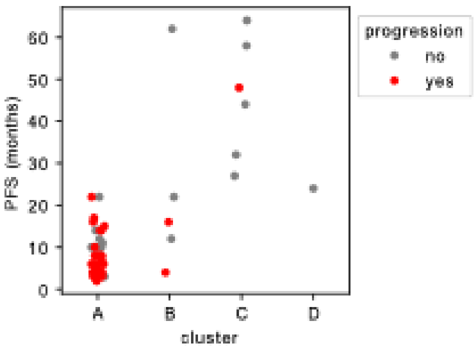

Minor Points

1. Category "I" represents samples that cannot be clearly assigned to groups G or H, but the main text gives little explanation of how these intermediate cases are handled during DMTree construction (e.g. lines 103-104). It is unclear whether they influence clustering or are treated largely as noise. Although the Methods describe a procedure, the narrative is insufficient, and additional clarification in the main text would be helpful.

Response: We thank the reviewer and agree that our narrative did not sufficiently explain how category "I" is handled. In metilene3, G/H/I is a local, per-DMR assignment (from continuous methylation distances). However, "I" denotes ambiguous/near-tie evidence, not noise. During DMTree construction, the samples in "I" are assigned to the smaller group (less samples) per DMR. However, due to the accumulation of clustering evidence across DMRs, only recurrent groups impact DMTree. We added a paragraph to the main text stating how "I" is handled during the splits.

2. The paper does not describe how key parameters were set. For example, the criteria for finalizing clusters in the DMTree are mentioned, but the threshold values and their rationale are not provided. It is also unclear what significance or effect size thresholds were used for calling DMRs, as no explicit FDR or p-value cutoff is stated in the main text. Clear reporting of these details would be helpful.

Response: We thank the reviewer and agree that our parameter reporting was insufficiently explicit. We now state all key thresholds and defaults in the methods and mention them in the figure legends.

3. Metilene3 reports a very large number of DMRs in several analyses, which raises minor questions about how many of these regions have meaningful effect sizes or biological relevance. Aside from post-hoc motif analyses, the manuscript does not describe how important DMRs are prioritized. A brief explanation of how these large DMR sets should be interpreted or filtered would be helpful.

Response: This question, as well as the remarks about batch effects, motivated us to expand DMR testing by adding an ANOVA after the core metilene run for the supervised analysis. By this, weaker and more heterogeneous DMRs are filtered out. We additionally added a section to the documentation stating that the DMR length and delta are good sorting and filtering strategies.

4. The examples of “double-switch” DMR patterns at the TRAK1 and TXNRD1 loci provide key evidence for the biological insights enabled by metilene3’s local clustering approach (lines 318-338). Given their importance, it may be worth considering moving at least one representative example into the main figures to help readers appreciate the significance of these findings without relying solely on the Extended Data.

Response: We thank the reviewer for this very supportive and positive suggestion and moved the double-switch DMRs to the main figures.

5. Some terms and acronyms appear before being defined (for example, “MDS” for methylation distance score), which may confuse readers on first encounter.

Response: Our apologies for potentially having missed this. We checked the text to avoid these confusions. However, we think MDS first appears in the first paragraph of the results (line 106), where the abbreviation is introduced.

Reviewer #4 (Remarks on code availability):
I was not able to access the source code, so I could not assess whether it includes sufficient documentation (such as a README), installation instructions, or runnable scripts. Without access to the code, it is not possible to evaluate the reproducibility of the analyses or determine whether the code is a useful resource for the community. I recommend that the authors make the code publicly available to support transparency and reproducibility.

Response: We are very sorry that the reviewer had this problem. In our hands (in incognito mode), for three different test persons, as well as for reviewer #3 it seems that the access did work. Depending on the reviewer’s location, a VPN may be needed to access GitHub. To ease access, we additionally uploaded the code as reviewer material.

Reviewer #1 (Remarks to the Author):

While the authors have addressed the major issues related to the novelty of Metilene3 and its improved performance, the revised manuscript still does not provide new biological insights resulting from the use of this tool. With the claims regarding biological interpretation toned down, it remains unclear what new biological insights this study ultimately provides.

Response: We thank the reviewer for their feedback in the previous revision and for their appreciation of the newly presented results. Regarding the remaining point, we would like to clarify that the primary aim of this manuscript is to present a methodological contribution. Therefore, we feel that conducting the extensive wet-lab experiments required to validate novel biological findings would be beyond the intended scope of this work.

Reviewer #2 (Remarks to the Author):
I co-reviewed this manuscript with one of the reviewers who provided the listed reports. This is part of the Nature Communications initiative to facilitate training in peer training and to provide appropriate recognition for Early Career Researchers who co-review manuscripts.

Response: We thank the Early Career Researcher for their interest and time!

Reviewer #3 (Remarks to the Author):
The authors have addressed all my previous concerns. However, the benchmarking data should be made available in the GitHub repository or the supplementary materials to ensure research reproducibility and facilitate comparison with other studies.

Response: We thank the reviewer for their positive evaluation. All the benchmarking data and supplementary materials referred to in this manuscript and currently available via the supplementary zip archive will be published upon acceptance using an immutable Zenodo object.

Reviewer #4 (Remarks to the Author):
The revised manuscript has addressed most of my previous comments in a careful and thoughtful manner, and I appreciate the authors' efforts to improve the study. I have only two remaining points that, if clarified, would further strengthen the manuscript:

Response: We appreciate the reviewers' very helpful comments and feedback on the manuscript and revision.

Regarding Major Point 3: The additional simulations and DNA-mixing analyses are appreciated. However, it remains unclear how these results translate to real-world datasets. Could the authors provide a quantitative estimate of the minimal tumor purity required for reliable clustering, and clarify how performance degrades below this threshold?

Response: We thank the reviewer for this important suggestion. We concur that simulating different purities helps to understand the limits of metilene3. Since tumor purity varies across cancer types, we used the TCGA cohort to obtain realistic purity ranges, with the lowest mean purity being ~0.6 (LUAD).

Action taken: In addition to the current simulation (simulated set A, with a purity mean of 0.87, inspired by the TCGA-LGGGBM cohort), we added four simulated data sets B-E with purity means ranging from 0.8 to 0.5. Our results showed that DMTree accurately predicted sample groups when purity was at least 0.6. Samples from different groups could no longer be fully distinguished when purity fell below 0.6 (**Extended Data Fig. 3f, g**).

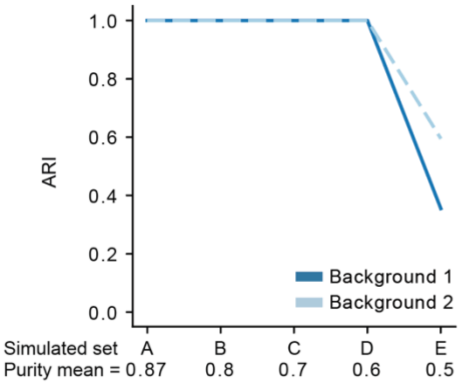

Extended Data Fig. 3g: *Recoverability of A to E using DMTree, under varying purity levels. Recoverability is defined as the ARI on samples from groups A-E. ARI: average Rand index.*

Regarding Major Point 4: The clarification of the DMTree framework is helpful. However, the basis for its improved robustness remains unclear. Could the authors clarify whether the performance gain is primarily driven by the use of DMR-level aggregation rather than the tree-based strategy itself?

Response: We thank the reviewer for their question regarding the underlying causes of metilene3's performance gain. Attributing the gain to either the aggregation or the tree-based strategy is not straightforward. This is because the DMTree is built on DMR-level aggregated data, and from that data, DMRs are 'selected' by the DMTree construction method. The final robustness of the result is a consequence of both procedures.

To still estimate the influence of the individual procedures, we extracted all DMRs selected during DMTree construction but applied a different clustering strategy. In this way, the alternative clustering method (hierarchical and k-means clustering) could use the same DMRs as those used in the respective DMTree.

Action taken: We performed the same benchmarking as in Figure 3d but replaced most variable CpGs by DMTree-DMRs for hierarchical clustering and k-means. Using the DMTree-DMRs with hierarchical and k-means clustering already improved robustness (**Extended Data Fig. 3e**). However, the robustness of neither method matches that of the complete DMTree approach, indicating that both DMR-level aggregation and the tree-based strategy contributed to the improved robustness.

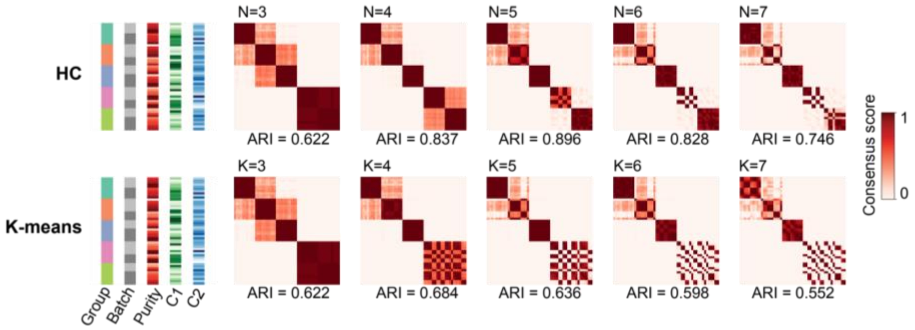

Extended Data Fig. 3e: *Recoverability of groups G1 and G2 using hierarchical clustering (HC), and K-means clustering using DMTree used DMRs, under varying proportions of group G1 and different clustering model parameters. Recoverability is defined as the ARI on samples from groups G1 and G2. ARI: average Rand index.*
